# Retinoic Acid Reprograms Mast Cells Toward a Proinflammatory State to Enhance Antitumor Immunity

**DOI:** 10.1002/advs.202509340

**Published:** 2025-11-27

**Authors:** Lizao Zhang, Siqi Ren, Yuhui Li, Rongxi Chen, Ling Qiu, Yongmei Tan, Suling Chen, Huiqian Wu, Lianxi Mai, Xiao Tan, Xin Liu, Peisheng Liang, Shijia Kuang, Liansheng Wang, Jingkang Liu, Jintao Li, Yanyan Li, Qiuping Xu, Yongzhi Su, Yuehai Luo, Binyan Wu, Zijing Huang, Haotian Cao, Qunxing Li, Jinsong Li, Tianjun Lan

**Affiliations:** ^1^ Department of Oral and Maxillofacial Surgery Sun Yat‐sen Memorial Hospital of Sun Yat‐sen University Guangzhou 510120 China; ^2^ Guangdong Provincial Key Laboratory of Malignant Tumor Epigenetics and Gene Regulation Guangdong‐Hong Kong Joint Laboratory for RNA Medicine Medical Research Center Sun Yat‐sen Memorial Hospital Sun Yat‐sen University Guangzhou 510120 China; ^3^ Department of Pathology Sun Yat‐sen Memorial Hospital Sun Yat‐sen University Guangzhou 510120 China; ^4^ Department of Stomatology Affiliated Hospital of Hangzhou Normal University Hangzhou 310015 China; ^5^ Department of Stomatology Shunde Hospital Southern Medical University (The First People's Hospital of Shunde) Foshan 528308 China; ^6^ Guanghua School of Stomatology Hospital of Stomatology Guangdong Province Key Laboratory of Stomatology Sun Yat‐sen University Guangzhou 510095 China; ^7^ Medical Research Center Sun Yat‐sen Memorial Hospital Sun Yat‐sen University Guangzhou 510120 China; ^8^ School of Mathematics and Big Data Foshan University Southern Medical University Foshan 528000 Guangzhou 510000 China; ^9^ Department of Endodontics Stomatological Hospital Southern Medical University Guangzhou 510000 China

**Keywords:** cancer immunotherapy, Mast cells, pan‐cancer, retinoic acid, single‐cell RNA sequencing

## Abstract

Mast cells play complex and context‐dependent roles within the tumor microenvironment, yet their molecular characteristics and functional diversity across human cancers remain poorly defined. In this study, single‐cell RNA sequencing and spatial transcriptomics data are integrated to comprehensively map the transcriptional and spatial heterogeneity of mast cells across ten cancer types. A distinct proinflammatory mast cell (PMC) population is identified, characterized by strong antigen‐presenting features and immune‐activating potential. Mechanistic analyses show that retinoic acid (RA) signaling drives the polarization of PMCs through activation of RARα, which promotes CIITA‐mediated MHC‐II expression, CXCL16 secretion, and T cell recruitment and activation. Across multiple cancer types, tumors with higher PMC abundance are associated with more favorable clinical outcomes. These findings reveal the pivotal role of RA–RARα–CIITA signaling in mast cell reprogramming and suggest that pharmacologic induction of proinflammatory mast cells may represent a promising approach to enhance antitumor immunity.

## Graphical abstract

Proinflammatory mast cells represent an MHC‐IIhigh, cytokine‐producing mast cell subset associated with improved survival and enhanced responses to anti‐PD‐1 therapy. Retinoic acid drives their polarization, enabling antigen uptake and presentation, and T cell recruitment and activation, collectively promoting adaptive anti‐tumor immunity.

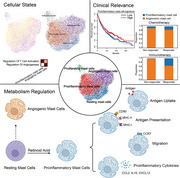

## 1 Introduction

Mast cells were first reported by Paul Ehrlich in 1877.^[^ ^1^ ^]^ These tissue‐resident cells are characterized by their abundance of granules rich in histamine and heparin. Traditionally, mast cells have been recognized for their pivotal role in allergic diseases. However, recent research has unveiled their involvement in chronic inflammatory diseases and cancer.

In cancer immunology, the dual role of mast cells as either suppressors or protectors remains a paradox. Within the tumor microenvironment, activated and degranulated mast cells can recruit innate immune cells, such as neutrophils and macrophages, as well as acquired immune cells like T and B cells, thus orchestrating an antitumor immune response.^[^ ^2^, ^3^, ^4^, ^5^ ^]^ Conversely, mast cells can also promote tumor progression by facilitating angiogenesis and metastasis.^[^ ^6^ ^]^ Increased mast cell accumulation in tumors has been correlated with tumor progression, immune evasion, therapy resistance, and poor prognosis across several cancer types, including breast cancer,^[^ ^7^ ^]^ prostate cancer,^[^ ^8^ ^]^ and renal cell carcinoma.^[^ ^9^ ^]^ However, recent studies have demonstrated that mast cell‐derived TNF can amplify dendritic cell function and T cell priming.^[^ ^10^ ^]^ High ratios of TNF^+^/ VEGFA^+^ mast cells have been reported to be associated with better prognosis in nasopharyngeal cancer.^[^ ^11^ ^]^ These findings suggest that mast cells exhibit different phenotypes and drive either pro‐ or anti‐tumor effects depending on the tissue or disease context.

Given their presence in diverse tumor types, ability to influence immune cell recruitment, and association with both tumor progression and immune activation, mast cells have emerged as an underexplored but functionally versatile component of the tumor microenvironment. Yet, their molecular heterogeneity, spatial organization, and tumor‐specific phenotypes across cancer types remain largely unknown. A pan‐cancer approach provides a unique opportunity to systematically dissect these features and identify common or distinct mast cell programs that may underlie their dual roles in tumor biology. Similar integrative single‐cell RNA sequencing strategies have been successfully applied to dissect the tumor microenvironment across multiple cell lineages, including T cells,^[^ ^12^ ^]^ B cells,^[^ ^13^ ^]^ myeloid cells,^[^ ^11^ ^]^ fibroblasts,^[^ ^14^ ^]^ and endothelial cells.^[^ ^15^ ^]^ Using a comparable framework, we systematically mapped mast cell diversity across human cancers to uncover their shared and cancer type‐specific programs.

In this study, we combined single‐cell RNA sequencing (scRNA‐seq) and spatial transcriptomics to profile the transcriptional diversity and spatial heterogeneity of human mast cells across 622 samples collected from 399 patients encompassing ten cancer types, including paired uninvolved normal tissues and metastases from selected cancers. Our comprehensive analysis elucidates the heterogeneity, phenotypes, functions, spatial distributions, and metabolic activities of tumor‐infiltrating mast cells. We discovered a distinct subset of proinflammatory mast cells with antigen‐presenting properties, which were associated with improved prognosis and enhanced response to anti‐PD‐1 therapy. Building upon these findings, we investigated potential regulators of proinflammatory mast cell polarization and identified retinoic acid (RA) as a functional inducer. Through transcriptomic and functional assays, we demonstrated that RA enhances the antigen‐presenting capacity of mast cells and promotes T cell activation. Together, our work bridges large‐scale single‐cell discovery with mechanistic validation, revealing both the heterogeneity and plasticity of mast cells and underscoring the therapeutic potential of targeting mast cell polarization in cancer immunotherapy.

## 2 Experimental Section

### 2.1 scRNA‐seq Data Collection

The scRNA‐seq data were obtained from various public studies.^[^ ^16^, ^17^, ^18^, ^19^, ^20^, ^21^, ^22^, ^23^, ^24^, ^25^, ^26^, ^27^, ^28^, ^29^, ^30^, ^31^, ^32^, ^33^, ^34^, ^35^, ^36^, ^37^, ^38^, ^39^ ^]^ The data accession numbers and references for these public datasets are provided in Table S1 (Supporting Information). Detailed clinical information on patients and samples is provided in Table S2 (Supporting Information).

### 2.2 Sample Preparation, scRNA‐seq, and Library Construction of In‐House Data

A total of eight tumor biopsy samples were collected during routine clinical procedures, serving as the discovery cohort, and subsequently stored in MACS Tissue Storage Solution (Miltenyi, Bergisch Gladbach, Germany). The samples were enzymatically digested using the gentleMACS Tumor Dissociation Kit (Miltenyi) at 37 °C for 60 min following the manufacturer's instructions. After digestion, the dissociated cells were filtered through a 40 µm cell strainer (Biosharp, Hefei, Anhui, China) and centrifuged at 300 × *g* for 10 min. The cell pellet was then suspended in red blood cell lysis buffer, followed by resuspension in phosphate‐buffered saline (PBS, Gibco, Carlsbad, CA, USA) with 2% fetal bovine serum (FBS, ExCell, Shanghai, China) after two washes with PBS.

Single‐cell RNA sequencing (scRNA‐seq) libraries were constructed in accordance with the manufacturer's guidelines. Briefly, after an additional PBS wash containing 0.04% bovine serum albumin (BSA, Invitrogen, Carlsbad, CA, USA), the cell density was determined. Approximately 2 × 10^5 cells were then loaded onto the 10x Genomics GemCode Single‐Cell Instrument (10x Genomics, San Francisco, CA, USA), which generates single‐cell Gel Bead‐In‐Emulsion (GEMs). Libraries were prepared and sequenced using cDNA generated with the Chromium Next GEM Automated Single Cell 3′ cDNA Kit v3.1 (10x Genomics). Upon GEM dissolution, primers containing an Illumina R1 sequence (read 1 sequencing primer), a 16 nt 10x Barcode, a 10 nt Unique molecular identifier (UMI), and a poly‐dT primer sequence were released and combined with the cell lysate and Master Mix. Barcoded full‐length cDNAs were reverse‐transcribed from polyadenylated mRNA. Silane magnetic beads were employed to remove residual biochemical reagents and primers from the post‐GEM reaction. The barcoded cDNAs were then amplified using quantitative real‐time PCR (qRT‒PCR) to generate sufficient material for library construction. Finally, single‐cell RNA libraries were sequenced on an Illumina HiSeq X‐Ten platform, generating 150 bp paired‐end reads.

### 2.3 scRNA‐seq Data Processing

Scanpy (RRID:SCR_018139) was used to analyze scRNA‐seq data. Briefly, cells with low complexity libraries (<500 genes), likely dying or apoptotic cells (>10% of transcripts are derived from the mitochondria), and cells with high‐complexity libraries (>5000 genes) were removed. Genes expressed in fewer than ten cells were also excluded. Additional filters included doublet removal using Srublet. After quality control, following the standard protocol of scanpy (RRID:SCR_018139), the count data were normalized and logarithm‐transformed. The top 2000 highly variable genes were selected for downstream analysis. After completing principal component analysis, 50 principal components were selected to compute the neighborhood relations. Batch effects were corrected using BBKNN (RRID:SCR_022807). Representative markers were used to annotate clusters.

### 2.4 Proportion Analysis

**Figure 1:**
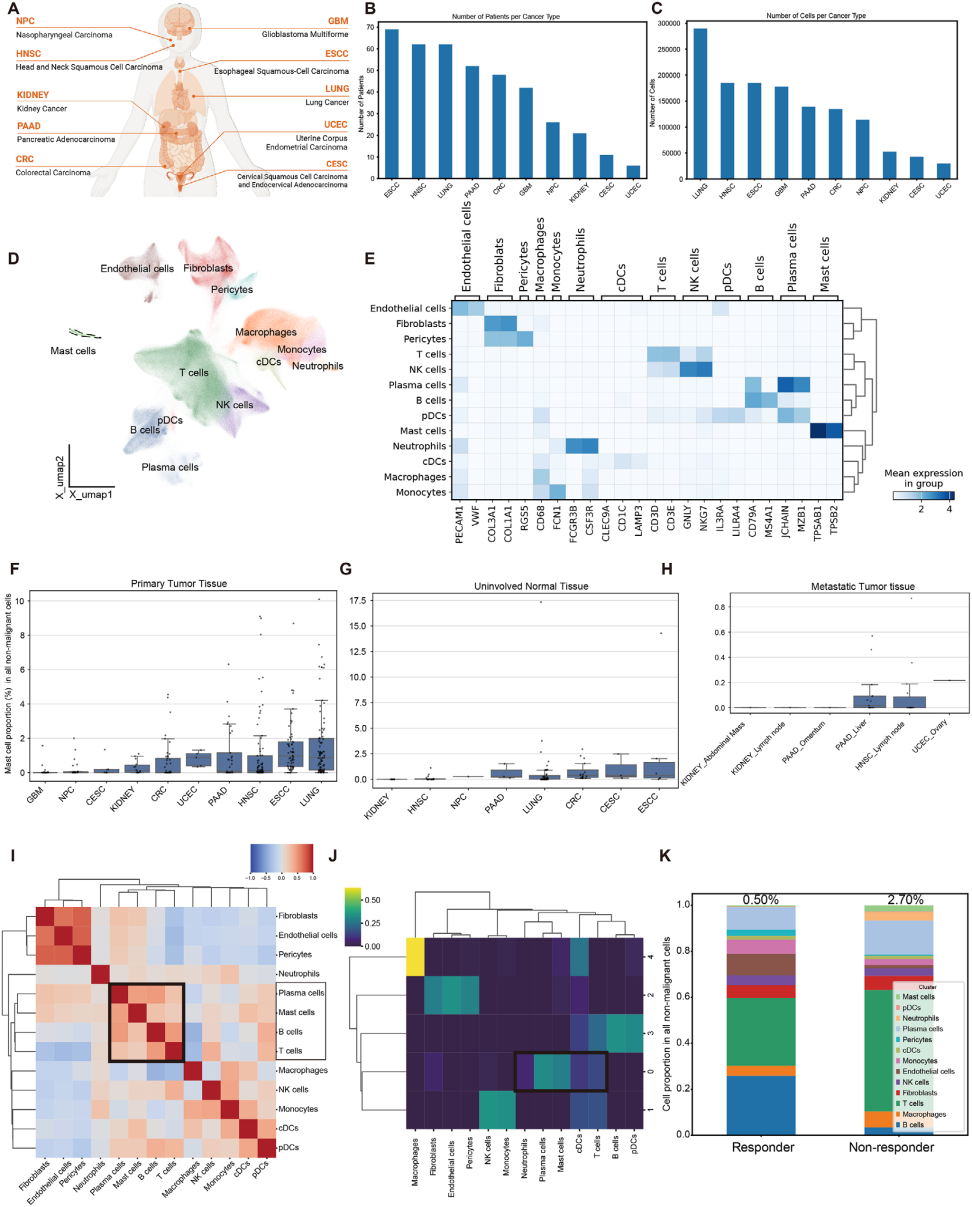
Pan‐cancer analysis of mast cells. A) Schematic overview of the cancer types included in this study (created with BioRender.com). B) Bar graph summarizing the number of patients collected by cancer type. C) Bar graph summarizing the number of cells collected by cancer type. D) UMAP visualization of the 13 major cell clusters identified in the study. E) Matrix plot displaying marker genes for the 13 major cell clusters. F) Boxplot showing the proportion of mast cells among all non‐malignant cells in primary tumor tissues across different cancer types. The figures on the bars indicate the proportion of mast cells. G) Boxplot showing the proportion of mast cells among all non‐malignant cells in uninvolved normal tissues across different cancer types. The figures on the bars indicate the proportion of mast cells. H) Boxplot showing the proportion of mast cells among all non‐malignant cells in metastatic tumor tissues across different cancer types and metastatic sites. The figures on the bars indicate the proportion of mast cells. I) Heatmap showing the correlation between the 13 major cell clusters. J) Heatmap displaying the clustering of NMF cellular programs. K) Stacked bar graph showing the proportion of mast cells among all non‐malignant cells in primary tumor tissues between responders and non‐responders to immunotherapy. The figures on the bars indicate the proportion of mast cells.

**Figure 2:**
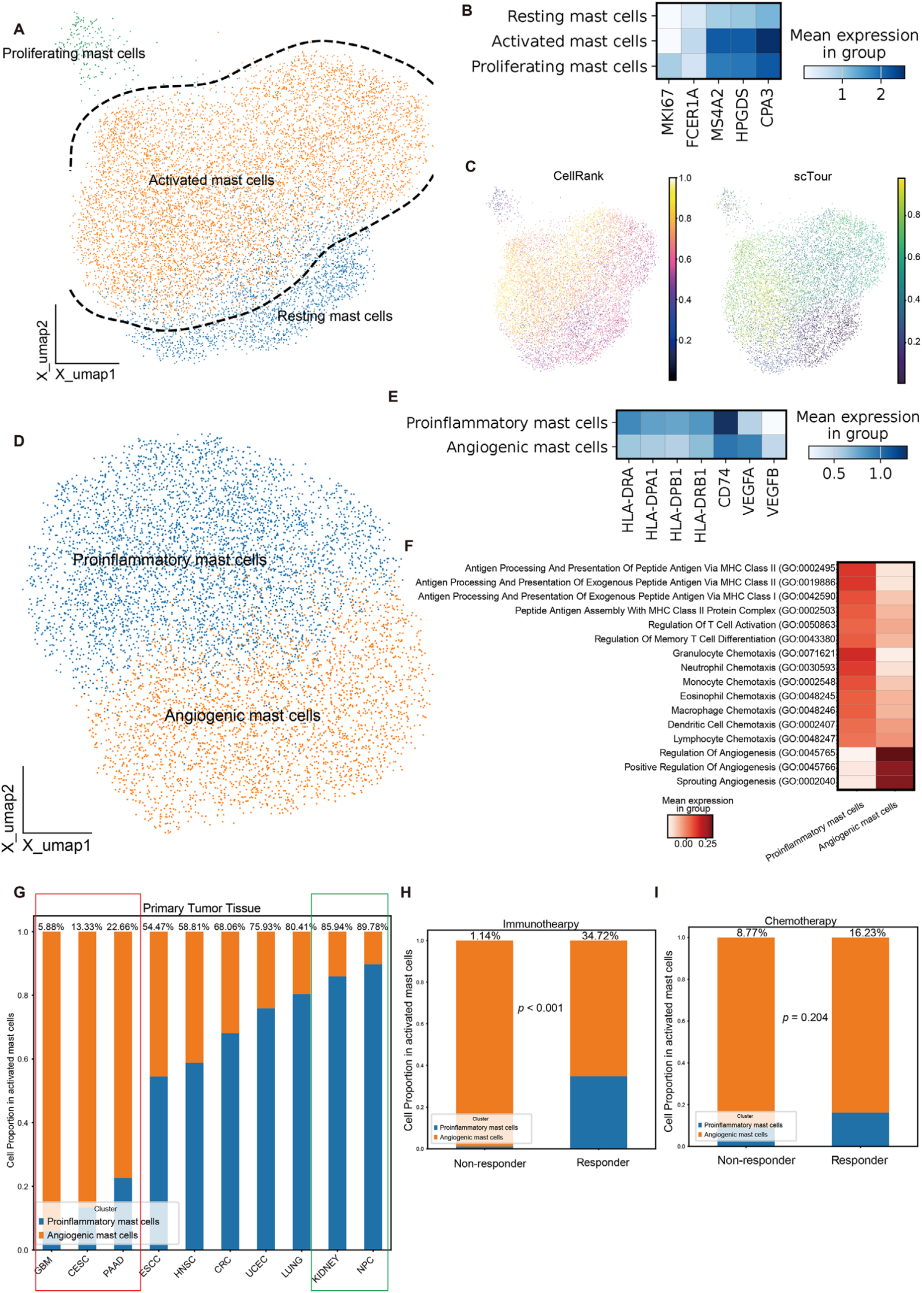
Transcriptional signatures of mast cells across cancers. A) UMAP visualization of the three mast cell subclusters. B) Matrix plot displaying marker genes for the three mast cell subclusters. C) UMAP visualization showing the pseudotime score calculated by CellRank (left) and scTour (right). D) UMAP visualization of proinflammatory mast cells and angiogenic mast cells, the latter corresponding to the previously reported VEGFA⁺ mast cell population.^[^ ^11^ ^]^ E) Matrix plot displaying marker genes for proinflammatory mast cells and angiogenic mast cells. F) Heatmap showing the pathways enriched in proinflammatory mast cells and angiogenic mast cells. G) Stacked bar graph showing the proportion of proinflammatory mast cells among activated mast cells in primary tumor tissues across different cancer types. The figures on the bars indicate the proportion of proinflammatory mast cells. H) Stacked bar graph showing the proportion of proinflammatory mast cells among activated mast cells in primary tumor tissues between responders and non‐responders to immunotherapy. The figures on the bars indicate the proportion of proinflammatory mast cells. I) Stacked bar graph showing the proportion of proinflammatory mast cells among activated mast cells in primary tumor tissues between responders and non‐responders to chemotherapy. The figures on the bars indicate the proportion of proinflammatory mast cells.

In this study, the cellular abundance of mast cells was calculated as the ratio of mast cell count to the total cell count (**Figure** **1**F–H), immune cell count (Figure S2A–C, Supporting Information), and myeloid cell count (Figure S2D–F, Supporting Information) from different tissue types. The cellular abundance of resting, activated, and proliferating mast cells was calculated as the ratio of the specific mast cell type count to the total mast cell count (Figure S3B–D, Supporting Information). The cellular abundance of proinflammatory and angiogenic mast cells was calculated as the ratio of the specific mast cell type count to the activated mast cell count (Figure 2G, Figure S3G,H, Supporting Information). For the immunotherapy cohort, due to the small number of mast cells, patients from GSE205506 and GSE207422 were analyzed together. Patients with PCR or MPR were considered responders, while those with non‐PCR or NMPR were considered non‐responders. GSE205013 was used as the chemotherapy cohort, with patients achieving PR considered responders, and those with SD or PD considered non‐responders.

### 2.5 Single‐Cell Pathway Analysis

AUCell (RRID:SCR_021327) algorithm integrated in OmicVerse was used to conduct pathway analysis on mast cell subclusters. Briefly, the expression matrix was normalized and logarithm‐transformed. The ‘GO_Biological_Process_2023′ gene set was selected to conduct enrichment analysis.

### 2.6 NMF Analysis for Cellular Program Identification

Cellular program identification was inspired by Chen et al.^[^ ^40^ ^]^ and conducted using cNMF (RRID:SCR_025495). Briefly, to identify cellular programs in the tumor microenvironment, first, the proportion of all cell types in each tumor sample was calculated, obtaining the abundance matrix ***V*** (cell type × sample). cNMF (RRID:SCR_025495) was used to factorize the abundance matrix ***V*** into two non‐negative low‐rank matrices: ***𝑉*** = ***𝑊𝐻***, the ***𝑊*** matrix documented the loading of the cell type on the corresponding factor, coefficient matrix ***𝐻*** reflected the activity score of the sample in the estimated NMF factor. 5 was used as the factorization rank based on the stability and error. Each NMF factor was regarded as a cellular program. Hierarchical clustering was then performed with the method of single linkage and Euclidean distance on the ***W*** matrix to detect the representative cell types for each cellular program.

### 2.7 Cellular Interaction Analysis

CellphonDB was used to investigate cellular interactions. For the cell–cell interactions between each mast cell subset and lymphoid cells, the predicted ligand–receptor pairs with *p*‐values < 0.05 were extracted for counting and presentation in the figures.

### 2.8 Trajectory Analysis

CellRank (RRID:SCR_022827) and scTour were used to investigate cell differentiation. For CellRank (RRID:SCR_022827) analysis, the CytoTRACE (RRID:SCR_022828) kernel was selected.

### 2.9 Single‐Cell Metabolism Analysis

The scMetabolism R package was used to conduct metabolism analysis on our data.

### 2.10 TCGA Data Analysis

The Cancer Genome Atlas (RRID:SCR_003193) Pan‐Cancer expression data and clinical data were downloaded on the UCSC Xena website (https://xenabrowser.net/). Only samples from solid tumors were kept (LAML and DLBC were filtered out). The mast cell infiltration score was calculated using the CIBERSORT (RRID:SCR_016955) algorithm. The absolute mode was chosen to calculate mast cell infiltration for inter‐sample comparison. The mast cell infiltration score was calculated as the sum of the resting mast cell score and the activated mast cell score. The resting mast cell signature, activated mast cell signature, proliferating mast cell signature, proinflammatory mast cell signature, and angiogenic mast cell signature were calculated using the PCA method in IOBR (RRID: SCR_025619) package. Briefly, differently expressed genes were calculated between these groups, and the highly expressed genes (adjusted *p*‐value< 0.05, log2FC> 0) in each cluster were used as signature genes. The expression of each gene in a signature was first *z*‐score transformed. Then, a principal component analysis was performed, and principal component 1 was extracted to serve as the gene signature score. Overall survival (OS) and OS time (days) were used in survival analysis. The survminer package was used to select the best cutoff points and plot Kaplan–Meier plots.

### 2.11 Patient Cohorts

Three cohorts (HNSC, CRC, and lung cancer) were analyzed from Sun Yat‐Sen Memorial Hospital, Sun Yat‐Sen University. In the HNSC cohort, 50 patients underwent neoadjuvant anti‐PD‐1‐based immunochemotherapy, receiving a combination of albumin‐bound paclitaxel, carboplatin, and an anti‐PD‐1 antibody (Sintilimab, Pembrolizumab, or Nivolumab). Patients were included based on the following criteria: 1) histologically confirmed diagnosis of HNSCC; 2) clinical stage II–IV disease as defined by the 8th edition of the American Joint Committee on Cancer (AJCC) guidelines; and 3) no prior history of anticancer therapy. Patients were excluded if they had 1) incomplete clinical or pathological data; 2) a history of other malignancies or concurrent cancers; 3) received prior systemic therapy, radiotherapy, or surgery for HNSCC before the neoadjuvant treatment; 4) active autoimmune diseases or conditions requiring long‐term immunosuppressive therapy; or 5) serious comorbidities that could interfere with the assessment of treatment response or survival outcomes. Patients were then categorized into two groups based on their pathological response: responders (pathological complete response [pCR] or major pathological response [MPR]) and non‐responders (partial pathological response [pPR] or no pathological response [pNR]).

The CRC cohort included 55 patients receiving surgery and adjuvant chemotherapy, who were divided into two groups based on overall survival: long‐term survivors (OS > 5 years) and short‐term survivors (OS < 5 years). In the CRC cohort, patients were retrospectively enrolled based on the following criteria: (1) histologically confirmed diagnosis of colorectal cancer (CRC); (2) availability of complete clinical information; (3) receipt of standard treatment with adequate follow‐up data to assess overall survival (OS). Patients were excluded if they had incomplete clinical or follow‐up data, a history of other malignancies, concurrent cancers, or received non‐standard or experimental therapies.

In the lung cancer cohort, 32 patients received neoadjuvant anti‐PD‐1‐based immunochemotherapy, including albumin‐bound paclitaxel, carboplatin, and an anti‐PD‐1 antibody (Camrelizumab or Tislelizumab). Eligible patients met the following inclusion criteria: (1) histologically confirmed diagnosis of non‐small cell lung cancer (NSCLC), including squamous carcinoma and adenocarcinoma; (2) clinical stage II–IV disease as defined by the 8th edition of the TNM staging system; (3) no prior systemic therapy or radiotherapy before neoadjuvant treatment; and (4) availability of complete clinical, pathological, and radiographic response data. Patients were excluded if they had 1) small cell lung cancer (SCLC) or other histological subtypes; 2) incomplete clinical or radiographic data; 3) prior or concurrent malignancies; 4) history of autoimmune diseases requiring systemic immunosuppression; or 5) severe comorbidities that could interfere with treatment or outcome assessment. These patients were classified into two groups based on radiographic response: responders (CR + PR) and non‐responders (SD + PD). Detailed patient characteristics are provided in Table S3 (Supporting Information).

Additionally, tissue microarrays purchased from Shanghai Outdo Biotech were used, including two microarrays corresponding to two cohorts (CRC, #HColA180Su14; and lung cancer, #HLugA180Su12), and a tissue microarray of the HNSC cohort from Sun Yat‐Sen Memorial Hospital, Sun Yat‐Sen University, to investigate the prognostic significance of mast cells. These microarrays were used to analyze the correlation of mast cells with patient survival across different cancer types. Detailed patient characteristics are provided in Table S4 (Supporting Information).

### 2.12 Cell Culture and Treatment

The human mast cell line HMC‐1(RRID:CVCL_0003) was purchased and cultured in Iscove's Modified Dulbecco's Medium (IMDM) containing 10% fetal bovine serum (Gibco) and 1% penicillin/streptomycin (Meilunbio). CD4^+^ and CD8^+^ T cells were separated from healthy donor peripheral blood using EasySep Human CD4^+^ T Cell Isolation Kit (STEMCELL, # 17952) and EasySep Human CD8^+^ T Cell Isolation Kit (STEMCELL, # 17953) according to the manufacturer's instructions.

For HMC‐1 treatment, 2 × 10^5^ mL^−1^ HMC‐1 cells were seeded in 12‐well plates. The cells were then treated with 100 U mL^−1^ IFN‐γ (Propretech, #300‐02) (IFN‐γ group) or 100 U mL^−1^ IFN‐γ plus 50 nM RA (MeilunBio, #MB1302) (IFN‐γ + RA group) for 48 or 72 h. TTNPB (#HY‐15682), Bexarotene (#HY‐14171), AM580 (#HY‐10475), Adapalene (#HY‐B0091), Palovarotene (#HY‐14799), Ro 41‐5253 (#HY‐116248) were purchased from MCE.

For T cell co‐culture, 1 × 10^6^  mL^−1^ CD8^+^ or CD4^+^ T cells were seeded in 12‐well plates and activated with CD3/CD28 Microbeads (Miltenyi, #130‐050‐101 and #130‐093‐247) and 300U mL^−1^ IL‐2 (Propretech, #200‐02) treatment in the conditioned medium from HMC‐1. After 72 h, TNF‐α expression was analyzed by flow cytometry.

### 2.13 T Cell Migration Assays

To assess the chemotactic properties of HMC‐1, a 24‐well Transwell system with 5 µm pore size (Corning, Inc., NY, USA) was utilized. In the top chamber, a total of 2 × 10^5^ T cells in 200 µL of fresh medium (without FBS) were seeded. In the lower chamber, 600 µL conditioned medium of HMC‐1 with indicated treatments. After a 24‐h incubation period, the cells that migrated through the Transwell membrane were stained and analyzed by flow cytometry. Recombinant Human CXCL16 (#201‐16) was purchased from PrimeGene, and ML339 (#HY‐122197) was purchased from MCE.

### 2.14 Multi‐Color Immunohistochemistry (mIHC)

According to the manufacturer's instructions, the mIHC staining of 50 HNSCC patients, 55 CRC patients, and 32 LC patients was performed using Opal 7‐Color Kit (Cat#0004100100, Panovue). The antibodies all were purchased from Cell Signaling Technology or Abcam, as follow: MHC Class II (dilution:1:1000, Cell Signaling Technology Cat# 68258, RRID:AB_3099585), Mast cell Tryptase (dilution:1:100000, Abcam Cat# ab2378, RRID:AB_303023), VEGFA (dilution:1:100000, ab52917, Abcam), EpCAM (dilution:1:10000, Abcam Cat# ab213500, RRID:AB_2884975), CD8 (dilution:1:200, #85336, CST), CD4 (dilution:1:200, Abcam Cat# ab133616, RRID:AB_2750883) and CXCL13 (dilution:1:1000, ab246518, Abcam). For each staining, a secondary horseradish peroxidase‐conjugated antibody was incubated with a tyramide‐coupled fluorophore: Opal 780, Opal 690, Opal 620, Opal 570, Opal 520, and Opal 480, respectively. The selected field of mIHC images was captured by using Akoya Vectra Polaris, then the QuPath software was used to analyze the images for cell detection and classification, and mutiplexed analysis. The cell density (number of cells per mm2) of mast cells, proinflammatory mast cells, and angiogenic mast cells was determined for each tumor sample. In mIHC analyses, proinflammatory and angiogenic mast cells were defined as MHC‐II⁺ Tryptase⁺ and VEGFA⁺ Tryptase⁺ cells, respectively.

### 2.15 Flow Cytometry Analysis

After cell counting, around 2 × 10^6^ cell suspensions were stained with fluorophore‐conjugated antibodies during 30 min at 4 °C after blocking with Fc Receptor. The fluorescent‐labeled antibodies were as followed: FITC anti‐CD8 (1:100; BioLegend Cat# 980908, RRID:AB_2888883), APC anti‐CD4 (1:100; BioLegend Cat# 980812, RRID:AB_2820224), BV421 anti‐CD3 (1:100; BioLegend Cat# 317343, RRID:AB_2565848), FITC anti‐CD40 (1:100; BioLegend Cat# 334305, RRID:AB_1186056), APC/Cyanine anti‐HLA‐DR (1:100; BioLegend Cat# 307617, RRID:AB_493587), BV421 anti‐CD117 (Biolegend; 1:100; #562434), PE anti‐TNF‐α (1:200; BioLegend Cat# 502908, RRID:AB_315260), APC/Cyanine anti‐CCR7 (1:200; BioLegend Cat# 353211, RRID:AB_10915272).

### 2.16 Ovalbumin (OVA) Uptake Assay

2 × 10^5^ mL^−1^ HMC‐1 cells were seeded in 12‐well plates. The cells were then treated with 100 U mL^−1^ IFN‐γ (Propretech, #300‐02) (IFN‐γ group) or 100 U mL^−1^ IFN‐γ plus 50 nM RA (MeilunBio, #MB1302) (IFN‐γ + RA group) for 48 h.

For the OVA uptake assay, PE‐labeled OVA (OVA/PE) (Bioss, #bs‐0283P‐PE) was added to the HMC‐1 cells and incubated for 24 h. The percentage of PE‐positive cells was assessed by flow cytometry to evaluate the OVA uptake capacity of HMC‐1 cells.

### 2.17 Antigen Presentation Assay

Tumor samples were dissociated using the Tumor Dissociation Kit, Human (Miltenyi Biotec, #130‐095‐929) according to the manufacturer's instructions. Mast cells were isolated by fluorescence‐activated cell sorting (FACS) based on surface markers: FITC anti‐CD45 (1:100, BD Biosciences Cat# 555482, RRID:AB_395874), BV421 anti‐CD117 (1:100, BD Biosciences Cat# 562434, RRID:AB_11154222), and PE anti‐FCER1 (1:100, Thermo Fisher Scientific Cat# 12‐5899‐42, RRID:AB_10804885). The isolated mast cells were either left untreated (control group) or treated with 100 U mL^−1^ IFN‐γ (Propretech, #300‐02) and 50 nM RA (MeilunBio, #MB1302) (experimental group) for 48 h. Following treatment, both groups underwent antigen loading with a cytomegalovirus (CMV) pp65 peptide pool (STEMCELL, #100‐0668) according to the manufacturer's instructions before proceeding to co‐culture experiments.

Peripheral blood was collected from the same patient, and peripheral blood mononuclear cells (PBMCs) were isolated by density gradient centrifugation. From the PBMC fraction, T cells were separated using either the EasySep Human T Cell Isolation Kit (STEMCELL, #17951), EasySep Human CD4 T Cell Isolation Kit (STEMCELL, #17952), or EasySep Human CD8 T Cell Isolation Kit (STEMCELL, #17953), depending on the specific subset required for the experiment. Isolated monocytes from PBMCs were differentiated into immature dendritic cells (DCs) using 500 U mL^−1^ IL‐4 (ThermoFisher, #200‐04‐100UG) and 1000 ng mL^−1^ GM‐CSF (ThermoFisher, #300‐03‐100UG) for 4 days. On Day 5, immature DCs were stimulated with the CMV pp65 peptide pool (STEMCELL, #100‐0668) and further matured using a cytokine cocktail containing 10 ng mL^−1^ TNF‐α (ThermoFisher, #300‐01A‐100UG), 10 ng mL^−1^ IL‐1β (ThermoFisher, #200‐01B‐100UG), 1000 U mL^−1^ IL‐6 (ThermoFisher, #200‐06‐100UG), and 1 µg mL^−1^ PGE2 (STEMCELL, #72192).

To evaluate the antigen presentation capacity of mast cells, mast cells and T cells were co‐cultured, while a DC and T cell co‐culture was included as a positive control. T cell activation was assessed by flow cytometry using the expression of 4‐1BB (1:100, BD Biosciences Cat# 564091, RRID:AB_2722503) and TNF (1:200, BioLegend Cat# 502908, RRID:AB_315260) as activation markers.

### 2.18 Mast Cell, T Cell, and Tumor Organoid Co‐Culture

Tumor samples were collected and processed immediately for organoid culture. The tissue was first minced into small fragments and enzymatically digested using the Tumor Dissociation Kit, Human (Miltenyi Biotec, #130‐095‐929), following the manufacturer's instructions. The resulting cell suspension was filtered through a 70 µm cell strainer to remove debris and undigested tissue. The isolated cells were resuspended in cold Matrigel (Corning, #354234) and seeded into pre‐warmed culture plates, allowing the Matrigel to solidify at 37 °C for 20–30 min. After solidification, organoid culture medium was added, consisting of advanced DMEM/F12 (Gibco, #12634010) supplemented with GlutaMAX (Gibco, #35050061), B27 (Gibco, #17504044), N2 (Gibco, #17502048), EGF (PeproTech, #AF‐100‐15), Noggin (PeproTech, #250‐38), and R‐spondin 1 (PeproTech, #120‐38). The medium was changed every 2–3 days, and organoids were passaged every 7–10 days using mechanical dissociation and enzymatic digestion with TrypLE Express (Gibco, #12604013). Organoids were maintained under 37 °C, 5% CO_2_ conditions, and used in downstream experiments after 1–2 weeks of expansion.

The co‐culture protocol was adapted from Koh et al.’s study.^[^ ^41^ ^]^ Mast cells and T cells were isolated as previously described. Following IFN‐γ and RA treatment, mast cells were exposed to tumor organoid‐conditioned medium for 24 h. T cells were then added to the conditioned mast cells, and the mast cell‐T cell co‐culture was maintained for an additional 48 h.

After co‐culture, tumor organoids were harvested and combined with the mast cell‐T cell mixture. The resulting organoid‐mast cell‐T cell suspension was centrifuged, resuspended in Matrigel (Corning, #354234), and seeded as 30 µL Matrigel bubbles per well. These co‐cultures were overlaid with tumor organoid medium and incubated at 37 °C with 5% CO_2_ for 72 h.

Following incubation, the co‐culture mixtures were dissociated and analyzed by flow cytometry. T cell activation was assessed based on the expression of 4‐1BB (1:100, BD Biosciences Cat# 564091, RRID:AB_2722503) and TNF (1:200, BioLegend Cat# 502908, RRID:AB_315260). Additionally, EpCAM (1:100, BioLegend Cat# 324207, RRID:AB_756081)‐positive tumor cells were evaluated using an Annexin V‐FITC/PI apoptosis assay kit (Meilunbio, #MA0220) to assess mast cell‐mediated T cell cytotoxicity.

### 2.19 Bulk RNA‐seq Analysis

To comprehensively explore transcriptional differences after RA treatment. RNA‐seq was performed between HMC‐1 + IFN‐γ (control) group and HMC‐1 + IFN‐γ + RA (test) group with 3 replicates. The RNA‐seq was performed by SequMed BoTechnology Inc.

1) RNA extraction

RNA extraction from cells was used TRIzol (Invitrogen 15596026) and Direct‐zol RNA Miniprep (Zymo R2050).

2) RNA quantification and qualification

RNA purity and concentration were checked using the NanoDrop One(Thermo, USA)and Qubit 4.0 Fluorometer(Thermo, USA). RNA integrity was measured using the Qsep100 (Bioptic, China).

3) Library preparation for Transcriptome sequencing

Sequencing libraries were generated using the Hieff NGSUltima Dual‐mode mRNA Library Prep Kit(Cat#12309, China). A total amount of 0.2 µg RNA per sample was used as input material for the RNA sample preparations. The first step is to enrich mRNA by the Poly(A) method with mRNA Capture Beads(BOX‐I 12603‐A), after mRNA Purification and Fragmentation, the RNA fragments are concentrated and the homogeneity, followed by synthesis of the 1st strand cDNA and the 2nd strand cDNA, 3′ ends adenylated and 5′ ends repaired, dA‐tailing, Adapter Ligation, Library Amplication. At last, PCR products were purified (Hieff NGS DNA Selection Beads (Yeasen Cat#12601) and library quality was assessed on Qubit dsDNA HS Fluorometer(Thermo, USA) and the Bioptic Qsep100 system.

4) Sequencing

The library preparations were sequenced on MGI SEQ‐T7, and 150 bp paired‐end reads were generated.

5) Quality control

Raw data (raw reads) of fastq format were first processed through fastp v0.23.0. In this step, clean data (clean reads) were obtained by removing reads containing adapters, reads containing N, and low‐quality reads, reads less than 36 bp after trimming from raw data. At the same time, Q20, Q30, and GC content were calculated. All the downstream analyses were based on the clean data with high quality.

6) Reads mapping to the reference genome

Reference genome and gene model annotation files were downloaded from Ensembl website directly. An index of the reference genome was built using Hisat2 v2.2.1, and clean reads were aligned to the reference genome using Hisat2 v2.2.1 to generate .sam or .bam files containing the alignments.

7) Quantification of gene expression level

The gene expression level quantification was performed separately for each sample using the featureCounts v2.0.1 software in the subread package, and the read count of each gene was obtained. The results of all samples were combined to obtain the expression matrix of all samples. The FPKM (Fragments Per Kilobase of exon model per Million mapped fragments) and TPM (transcripts per kilobase million) of each gene were calculated based on the length of the gene and read count mapped to this gene.

8) Differential expression analysis

Differential expression analysis of two conditions/groups was performed using the DESeq2 v1.34.0 R package. DESeq2 provide statistical routines for determining differential expression in digital gene expression data using a model based on the negative binomial distribution. The resulting *p*‐values were adjusted using Benjamini and Hochberg's approach for controlling the false discovery rate. Genes with an adjusted *p*‐value <0.05 found by DESeq2 were assigned as differentially expressed.

The selection criteria for significantly differentially expressed genes were: |log_2_FC| > 1 and FDR < 0.05. The significantly differentially expressed genes were used for downstream enrichment analysis and gene set enrichment analysis (GSEA). The significantly differentially expressed genes were listed in Table S5 (Supporting Information).

### 2.20 Cytokine Profile

Cytokine profile of HMC‐1 after indicated treatment was performed using RayBio Human Cytokine array C3 (RayBio, #AAH‐CYT‐3) according to the manufacturer's instructions. The raw data were analyzed after background subtraction and positive control normalization.

### 2.21 Spatial Transcriptomic Analysis

Spatial transcriptomic datasets were obtained from GEO.^[^ ^42^ ^]^ The spatial transcriptomic data were analyzed by Tangram. To evaluate the spatial co‐localization of the proinflammatory mast cell signature, we performed T cell clustering and mapped the annotated single‐cell dataset onto the spatial transcriptomic dataset using Tangram. This approach allowed us to assess the colocalization of proinflammatory mast cells, CD8‐CXCL13, and CD4‐CXCL13, providing insights into their spatial distribution and potential interactions within the tissue microenvironment.

### 2.22 Digital Spatial Profiling (DSP)

Slide preparation, DSP, and subsequent data analysis were conducted following the standard operating procedures provided by GeoMx DSP (available online at https://nanostring.com). Primary tumor samples from six HNSC patients were collected via biopsy, immediately formalin‐fixed and paraffin‐embedded (FFPE), and sectioned into 5 µm thick slices. These patients had no prior history of anticancer therapy and received a treatment regimen of paclitaxel, carboplatin, and pembrolizumab, administered every 3 weeks for up to 6 weeks before surgery. The slides underwent preprocessing steps, including deparaffinization in xylene, rehydration in graded ethanol, target retrieval in Tris‐EDTA with heat, and RNA target exposure by proteinase K, before being loaded onto the GeoMx DSP instrument.

The GeoMx Cancer Transcriptome Atlas was employed, which contains over 1800 RNA targets for comprehensive profiling of the tumor microenvironment. Nucleic acid probes conjugated to photocleavable oligonucleotide tags were applied to the slides and incubated overnight. The slides were then scanned using the GeoMx DSP instrument (NanoString Technologies, Inc.) to capture high‐precision, 20x images, which served as the basis for regions of interest (ROI) selection. Upon exposure to UV light generated by the DSP instrument, the UV‐cleaved oligo tags corresponding to specific ROIs were released into a solution, collected into a plate, and subjected to PCR amplification (Biorad) using GeoMx Seq Code primers for downstream sequencing. Following pooling, purification, and quality control, the library was prepared and sequenced using the Illumina NovaSeq next‐generation sequencing (NGS) platform (Illumina, San Diego, CA, USA). After sequencing, raw reads were processed through the GeoMx NGS Pipelines to generate digital count conversion (DCC) files for further analysis.

The NGS readouts underwent two rounds of quality control (QC) (detailed in the Supporting Information). Segment QC excluded ROIs that did not meet quality criteria, while probe QC removed outlier probes and targets outside acceptable thresholds. The limit of quantitation (LOQ) was set as the geometric mean of negative probes multiplied by their geometric standard deviation, with a confidence threshold of 2.0. Of the 1813 detected genes, 1770 (97.6%) were above the LOQ in at least 1% of the ROIs. QC‐qualified data were then normalized using three methods: Q3, housekeeping genes, and negative probes. After reaching consensus among these normalization methods (detailed in the Supporting Information), Q3 normalization was chosen as recommended by the GeoMx DSP User Manual.

The proinflammatory mast cell score, exhausted CD8 T cell score, and cytotoxic CD4 T cell score were calculated using the PCA method in the IOBR package,^[^ ^43^ ^]^ as described previously. The exhausted CD8 T cell score and cytotoxic CD4 T cell score were calculated using markers from Chu et al.’s study.^[^ ^44^ ^]^

### 2.23 Statistical Analysis

Statistical significance was defined as *p* < 0.05. Statistical analyses were performed using R (V4.4.0), Python (V3.10.14), and GraphPad Prism (V10.0.3). Group comparisons were conducted using Student's *t*‐tests and Wilcoxon rank‐sum tests. Unless otherwise specified, bar plots were presented as mean ± standard deviation. Each experiment was repeated three or more times using biologically independent samples. Spearman's rho was used for correlation analyses. Survival analyses were conducted using log‐rank tests.

### 2.24 Ethics Approval and Consent to Participate

All surgical tumor tissues used in this study were obtained from patients treated at Sun Yat‐Sen Memorial Hospital. The blood samples were obtained from healthy volunteers. This study was approved by the Ethical Committee of Sun Yat‐Sen Memorial Hospital (SYSKY‐2024‐927‐01), and the informed written consent of all participants was obtained for research with the collection of tissue and blood samples.

### 2.25 Availability of Data and Materials

All single‐cell transcription data obtained in this study were publicly available data, the reference was available in Supplemental Table S1. The TCGA Pan‐Cancer expression data and clinical data are available in the Xena database (Batch effects normalized mRNA data, Pan‐Cancer Atlas Hub) (https://xenabrowser.net/). The in‐house scRNA‐seq dataset, spatial transcription data, and other data that support the findings of this study are available from the corresponding author upon reasonable request.

### 2.26 Code Availability

The code related to this study is publicly available at https://github.com/giuyoung/Mast‐cell.

## 3 Results

### 3.1 Pan‐Cancer Analysis of Mast Cells

We compiled publicly available scRNA‐seq data on mast cells from 10 cancer types and 8 non‐neoplastic/healthy tissue types, encompassing 622 samples from 399 individuals (Figure 1A–C; Figure S1A, B, Supporting Information).^[^ ^16^, ^17^, ^18^, ^19^, ^20^, ^21^, ^22^, ^23^, ^24^, ^25^, ^26^, ^27^, ^28^, ^29^, ^30^, ^31^, ^32^, ^33^, ^34^, ^35^, ^36^, ^37^, ^38^, ^39^ ^]^ As shown in Figure S1C (Supporting Information), batch effects were substantially reduced after correction, as evidenced by the comparison between the uncorrected data (left) and the batch‐corrected data (right). The detailed information was shown in Tables S1 and S2 (Supporting Information). Using graph‐based clustering, we identified 13 major clusters based on characteristic markers of different cell types, including endothelial cells, fibroblasts, pericytes, T cell, NK cells, B cells, plasma cells, plasmacytoid dendritic cells (pDCs), mast cells, neutrophils, conventional dendritic cells (cDCs), macrophages and monocytes (Figure 1D,E and Figure S2, Supporting Information). Overall, 9815 high‐quality mast cells were identified following rigorous quality control (Methods).

Mast cells were one of the rarest clusters at tumor sites, with their proportion remaining relatively stable across tumor types. For example, mast cells were largely absent in glioblastoma multiforme (GBM), but were relatively abundant in lung cancer (LUNG) and esophageal squamous cell carcinoma (ESCC). This suggests that mast cells preferentially infiltrate certain cancer types, potentially exhibiting diverse functions (Figure 1F). A similar distribution pattern was observed when assessing the proportion of mast cells among immune or myeloid cells (Figure S3A,D, Supporting Information). Further analysis revealed higher proportions of mast cells in tumor tissues across most cancer types (Figure 1G and Figure S3B,E, Supporting Information). However, we observed increased mast cell presence in adjacent normal cervix and colorectal tissues, indicating their function in these normal tissues. Interestingly, the proportion of mast cells was lower in metastatic tumor tissues compared to primary tumor tissues (Figure 1H and Figure S3C,F, Supporting Information).

Correlation analysis between the 13 clusters indicated that mast cells are more frequently grouped with lymphoid cells than myeloid cells (Figure 1I), suggesting a role in adaptive immunity. Additionally, non‐negative matrix factorization (NMF) analysis revealed a cellular program involving mast cells, plasma cells, and T cells (Figure 1J). Pan‐cancer analysis using TCGA data further indicated that mast cell infiltration positively correlates with T cells (*p* < 0.001), B cells (*p* < 0.001), and plasma cells (*p* < 0.001) (Figure S3G, Supporting Information). However, it is worth noting that the correlation coefficients observed in this analysis are relatively low (R = 0.04–0.18). While these correlations are statistically significant due to the large sample size, their biological relevance may be limited. Together, these results suggest that mast cells exhibit modest but consistent associations with lymphoid cells, which aligns with their potential involvement in adaptive immunity. Cell interaction analysis demonstrated that mast cells interact with lymphoid cells via cell adhesion molecules and cytokines, such as CXCL16‐CXCR6, TNF‐TNFRSF1B, IFNG‐Type‐II‐IFNR, and SEMA4D‐CD72 (Figure S3H, Supporting Information). These results indicated that mast cells may play an important role in regulating adaptive anti‐tumor immunity in the tumor microenvironment.

**Figure 3:**
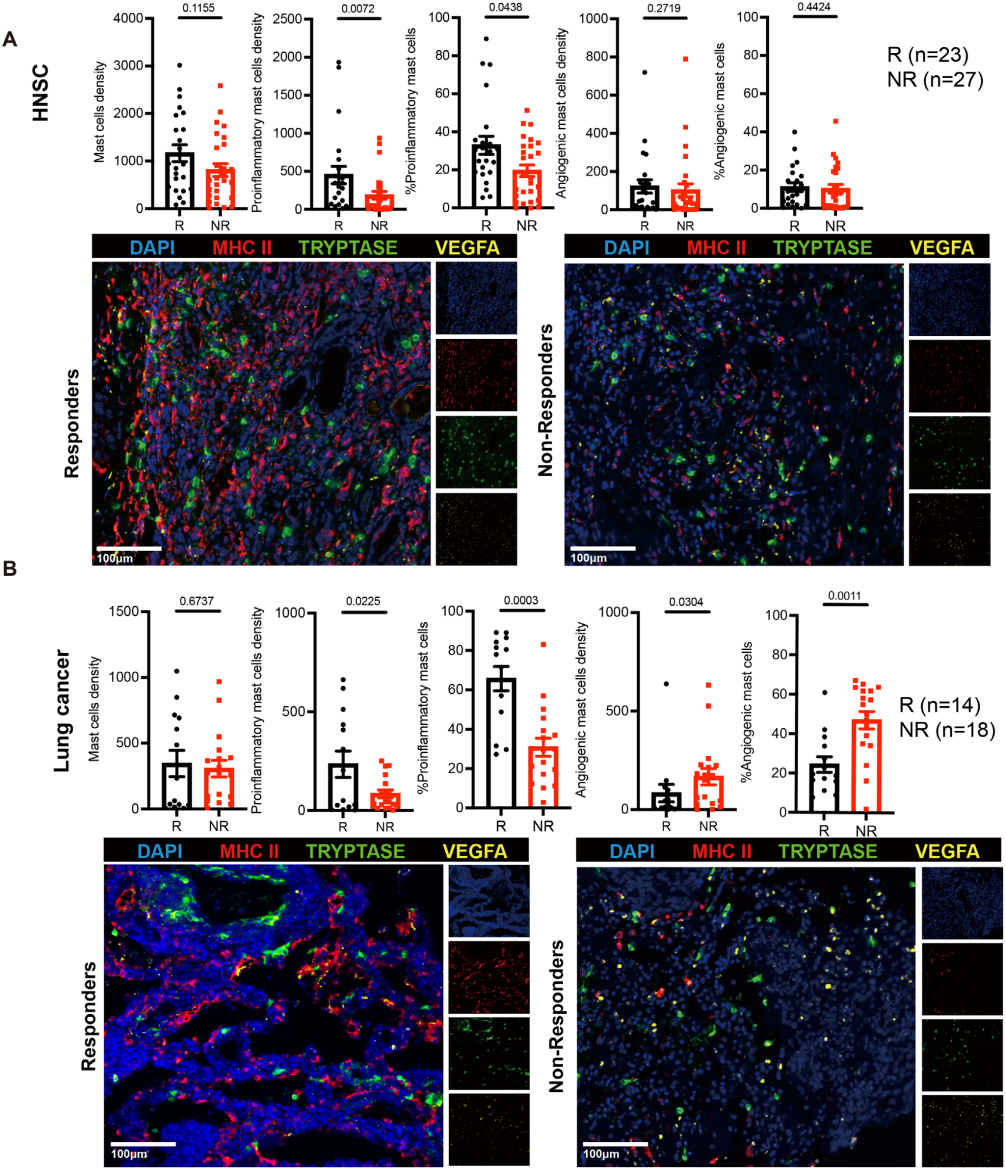
Proinflammatory mast cell infiltration was associated with better response to immunotherapy. A) Boxplots comparing mast cell density, proinflammatory mast cell density, proinflammatory mast cell proportion, angiogenic mast cell density, and angiogenic mast cell proportion between responders and non‐responders in the HNSC cohort receiving chemoimmunotherapy. Representative mIHC images of responders and non‐responders are shown. B) Boxplots comparing mast cell density, proinflammatory mast cell density, proinflammatory mast cell proportion, angiogenic mast cell density, and angiogenic mast cell proportion between responders and non‐responders in the lung cancer cohort receiving immunochemotherapy. Representative mIHC images of responders and non‐responders are shown.

Then, we analyzed the prognostic value of mast cells, though the mast cell infiltration was associated with worse survival in many cancer types such as GBM (*p* = 0.00022), CESC (*p* = 0.00025), and PAAD (*p* = 0.031), we found that mast cell infiltration was associated with better survival in kidney renal clear cell carcinoma (KIRC) (*p* = 0.00011) (Figure S3I, Supporting Information), underscoring the heterogeneity of mast cells across cancer types. In terms of immunotherapy, non‐responders exhibited higher mast cell proportions in a cohort receiving anti‐PD‐1 therapy (Figure 1K and Figure 3J, Supporting Information).

### 3.2 Transcriptional Signatures of Mast Cells Across Cancers

To decode the transcriptional signatures, we first clustered mast cells based on typical markers and trajectory analysis, identifying three major clusters: activated mast cells (expressing FCER1A, MS4A2, HPGDS, CPA3), proliferating mast cells (expressing MKI67), and resting mast cells with relatively low activation marker expression (**Figure** **2**A,B). These results were further validated by pseudotime analysis, resting mast cells had a lower pseudotime score, which indicated this cluster was located at the starting point of differentiation (Figure 2C). Pathway analysis revealed that activated mast cells were enriched for pathways related to mast cell activation, degranulation, and immune response (Figure S4A, Supporting Information). In most cancer types, activated mast cells constituted over 50% of the mast cells in primary cancer tissues, except in ESCC (Figure S4B, Supporting Information). In uninvolved normal tissues, CRC, HNSC, and ESCC had a relatively higher proportion of resting mast cells (Figure S4C, Supporting Information), while metastatic tumor tissues were predominantly composed of activated mast cells (Figure S4D, Supporting Information). Non‐responders to immunotherapy exhibited higher infiltration of activated mast cells (Figure S4E, Supporting Information, *p* = 0.01067).

Given that mast cells can polarize into distinct functional states, similar to macrophages, we performed subclustering analysis of activated mast cells and identified two main subsets: a proinflammatory population (highly expressing MHC‐II genes) and an angiogenic population (characterized by high VEGFA and VEGFB expression) (Figure 2D,E). Pathway analysis indicated that the former was enriched for antigen presentation and T‐cell activation, while the latter was associated with angiogenesis (Figure 2F). Importantly, this angiogenic subset corresponds to the previously reported VEGFA⁺ mast cell population.^[^ ^11^ ^]^ In primary tumor tissues, GBM, CESC, and PAAD had the lowest ratio of proinflammatory to angiogenic mast cells, while NPC and kidney cancer had the highest (Figure 2G). These results suggest that the positive prognostic value of mast cell infiltration in nasopharyngeal carcinoma (NPC)^[^ ^11^ ^]^ and KIRC may be attributed to a high proportion of proinflammatory mast cells, while the negative prognostic value in GBM, CESC, and PAAD may be due to a high proportion of angiogenic mast cells (Figure S3I, Supporting Information). In uninvolved normal tissues, proinflammatory mast cell infiltration was relatively lower in CESC and PAAD, suggesting that mast cell infiltration in these cancers may be linked to tumor progression (Figure S4F, Supporting Information). In HNSC metastatic tumor tissues, 100% of activated mast cells in metastatic lymph nodes were proinflammatory, indicating that the tissue microenvironment may influence mast cell polarization (Figure S4G, Supporting Information). In the immunotherapy scenario, responders exhibited significantly higher infiltration of proinflammatory mast cells (Figure 2H and *p* < 0.001). Similar results were shown when the proportion of proinflammatory and angiogenic mast cells was calculated in all mast cells (Figure S4H, Supporting Information, *p* < 0.001). Rodrigues et al.^[^ ^45^ ^]^ showed that PD‐1‐expressing mast cells can induce tolerogenic IDO^+^ dendritic cells, while Li et al.^[^ ^46^ ^]^ showed that PD‐1^+^ mast cells were associated with immunotherapy resistance in melanoma. However, we found proinflammatory mast cells had the lowest PD‐1 expression among mast cell subclusters (Figure S4I, Supporting Information), which indicated proinflammatory mast cells may play a positive role in immunotherapy. Besides, we found in a PAAD cohort receiving chemotherapy, responders also had relatively higher infiltration of proinflammatory mast cells (Figure 2I and *p* = 0.204). These findings indicate that proinflammatory mast cell infiltration may serve as a predictive marker for anti‐PD‐1 immunotherapy and chemotherapy.

### 3.3 Proinflammatory Mast Cell Infiltration was Associated with Better Response to Immunotherapy

To validate the predictive value of proinflammatory mast cell in anti‐PD‐1 immunotherapy, we performed multiplex immunohistochemistry (mIHC) on two clinical cohorts of patients receiving anti–PD‐1–based chemoimmunotherapy.

Detailed clinical information was shown in Table S3 (Supporting Information). In mIHC analyses, proinflammatory and angiogenic mast cells were defined as MHC‐II⁺ Tryptase⁺ and VEGFA⁺ Tryptase⁺ cells, respectively. The first cohort consisted of 50 HNSC patients receiving anti‐PD‐1‐based chemoimmunotherapy. As shown in **Figure** **3**A, the number (*p* = 0.0072) and proportion (*p* = 0.0438) of proinflammatory mast cells were significantly higher in responders. However, no significant difference was observed in the total number of mast cells, the number of angiogenic mast cells, or the proportion of angiogenic mast cells between responders and non‐responders. The second cohort involved 32 lung cancer patients who underwent anti‐PD‐1‐based immunochemotherapy, where we noted that both the number (*p* = 0.0225) and proportion (*p* = 0.0003) of proinflammatory mast cells were significantly higher in responders, with a notably lower number (*p* = 0.0304) and proportion (*p* = 0.0011) of angiogenic mast cells (Figure 3B). These findings underscore the predictive role of proinflammatory mast cell infiltration in immunotherapy response.

### 3.4 Proinflammatory Mast Cell Infiltration was Associated with Better Prognosis

To further validate the prognostic significance of mast cell subtypes, we analyzed three independent cohorts with available survival data (detailed in Table S4, Supporting Information). Across all three cohorts, we observed a significant association between total mast cell infiltration and worse survival outcomes. Specifically, proinflammatory mast cells were consistently correlated with better survival, whereas angiogenic mast cells were associated with worse survival (Figure S5 and S6A–C, Supporting Information). These findings align closely with our transcriptomic analysis results, reinforcing the robustness and reproducibility of our conclusions.

We further examined the spatial distribution of proinflammatory and angiogenic mast cells within the tumor microenvironment. Notably, proinflammatory mast cells tended to locate at the tumor–stroma interface (Figure S6D, Supporting Information), while angiogenic mast cells were preferentially located near CD34^+^ blood vessels (Figure S6E, Supporting Information). These spatial distinctions may help explain the conflicting prognostic significance of mast cells reported in different studies.

### 3.5 Retinoic Acid Regulates Proinflammatory Mast Cell Polarization

**Figure 4:**
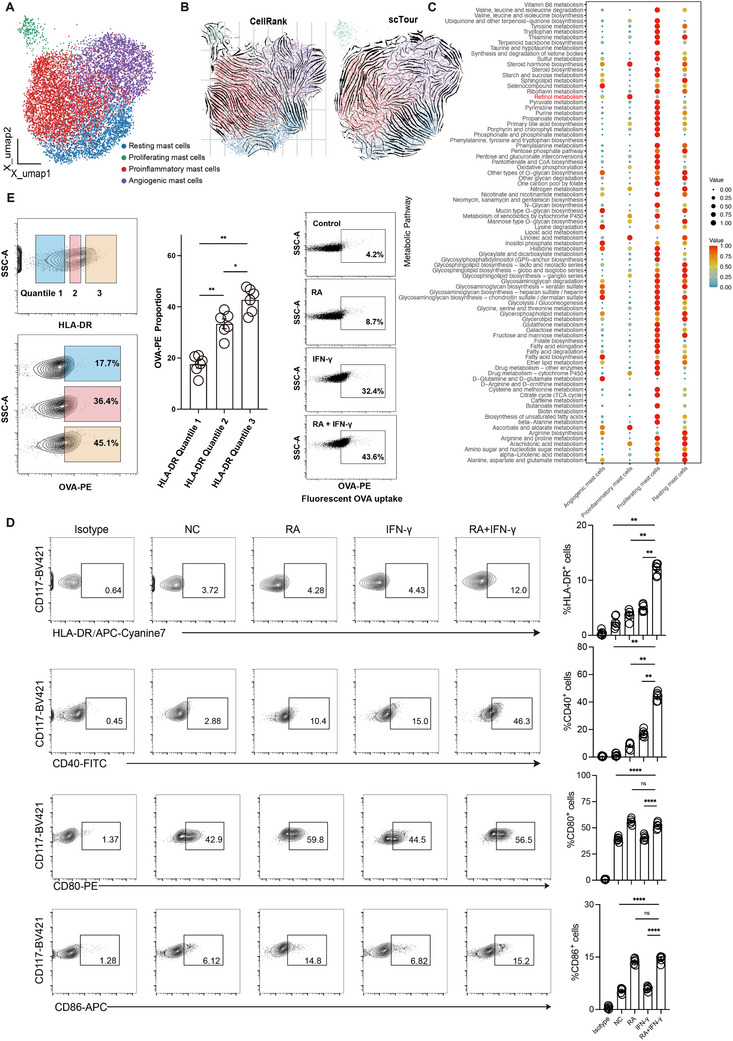
Retinoic acid regulates proinflammatory mast cell polarization. A) UMAP visualization of four mast cell subclusters, with proinflammatory mast cells and angiogenic mast cells projected onto activated mast cells. B) UMAP visualization showing the differentiation trajectory calculated by CellRank (left) and scTour (right). C) Dot plot displaying the enrichment of all metabolism‐related pathways in four mast cell subclusters. D) Flow cytometry analysis showing HLA‐DR, CD40, CD80, and CD86 expression after RA, IFN‐γ, and IFN‐γ + RA treatment for 48 h (*n* = 6). E) Flow cytometry analysis showing OVA‐PE uptake by HMC‐1 cells after RA, IFN‐γ, and IFN‐γ + RA treatment for 48 h (*n* = 6).

To investigate the polarization trajectory of mast cells, we projected proinflammatory mast cells and angiogenic mast cells onto all mast cells (**Figure** **4**A). Using CellRank and scTour, we found that after activation, resting mast cells could polarize into two distinct trajectories: proinflammatory mast cells and angiogenic mast cells (Figure 4B). Recent studies have shown the importance of metabolism in immune cell function.^[^ ^47^ ^]^ We further explored metabolic differences between mast cell subclusters and found that proinflammatory mast cells exhibited higher enrichment of retinol metabolism pathways (Figure 4C), suggesting that retinol metabolism plays a critical role in proinflammatory mast cell polarization. Given that RA is an active derivative of retinol,^[^ ^48^ ^]^ we tested its effect on mast cell polarization. As proinflammatory mast cells expressed high levels of MHC‐II molecules (Figure 2E), we firstly tested the MHC‐II expression after RA treatment. Consistent with a previous study,^[^ ^49^ ^]^ the mast cell line HMC‐1 did not express MHC‐II constitutively. As IFN‐γ was reported to activate mast cells and upregulate MHC‐II expression,^[^ ^49^, ^50^, ^51^ ^]^ we compared IFN‐γ + RA treatment to IFN‐γ treatment alone and RA treatment alone. Of note, when treated with IFN‐γ + RA, HLA‐DR expression was significantly upregulated (IFN‐γ versus NC, not significant, IFN‐γ + RA versus NC, *p* < 0.0001, IFN‐γ + RA versus IFN‐γ, *p* < 0.0001). (Figure 4D). Additionally, the co‐stimulatory molecule CD40 was also significantly upregulated following IFN‐γ + RA treatment (IFN‐γ versus NC, *p* < 0.0001, IFN‐γ + RA versus NC, *p* < 0.0001, IFN‐γ + RA versus IFN‐γ, *p* < 0.0001 (Figure 4D). In contrast, RA treatment alone had only a minor effect on HLA‐DR and CD40 expression (Figure 4D). Furthermore, the co‐stimulatory molecule CD80 and CD86 were significantly upregulated following IFN‐γ + RA treatment (IFN‐γ versus NC, not significant, IFN‐γ + RA versus NC, *p* < 0.0001, IFN‐γ + RA versus IFN‐γ, *p* < 0.0001). Notably, RA treatment alone was sufficient to induce CD80 and CD86 expression (Figure 4D). In addition, the expression of HLA‐DQ and CD74 (also known as the invariant chain, which functions as a chaperone for MHC class II molecules) was significantly upregulated following IFN‐γ + RA treatment (IFN‐γ versus NC, not significant, IFN‐γ + RA versus NC, *p* < 0.0001, IFN‐γ + RA versus IFN‐γ, *p* < 0.0001, Supplemental Figure S7A). Besides, HLA‐DR^high^ mast cells exhibited a significantly enhanced antigen uptake capability, with IFN‐γ + RA treatment inducing the highest OVA‐PE antigen uptake in mast cells (HLA‐DR Quantile 2 versus HLA‐DR Quantile 1, *p* = 0.0022, HLA‐DR Quantile 3 versus HLA‐DR Quantile 2, *p* = 0.0066, HLA‐DR Quantile 3 versus HLA‐DR Quantile 1, *p* < 0.0001) (Figure 4E). These results indicate that RA can enhance the antigen uptake and presentation capabilities of mast cells.

To comprehensively investigate the differences after IFN‐γ + RA treatment, we conducted bulk RNA‐seq comparing IFN‐γ + RA treatment to IFN‐γ treatment alone. Volcano plot revealed the upregulation of MHC‐II molecules such as HLA‐DRA, HLA‐DQB1, HLA‐DMB, and CD74, along with cytokines and chemokine receptors like CCL2, CXCL12, IL4, IL15, CSF2, and CCR7 in the IFN‐γ + RA treatment group (Figure S7B, Supporting Information). Pathway enrichment analysis showed that in addition to RA‐related pathways, pathways related to antigen presentation, T cell activation, chemotaxis, and cytokine production were also enriched in the IFN‐γ + RA treatment group (Figure S7C, Supporting Information). These findings were further validated in HMC‐1 cells, where flow cytometry confirmed the significant upregulation of CCR7 (IFN‐γ versus NC, *p* < 0.0001, IFN‐γ + RA versus NC, *p* < 0.0001, IFN‐γ + RA versus IFN‐γ, *p* < 0.0001), suggesting that RA may regulate mast cell migration and homing (Figure S7D, Supporting Information).

To validate these findings in primary cells, primary mast cells were isolated from human tumor tissues using CD45, CD117, and FCεRI as sorting markers (Figure S8A, Supporting Information). When treated with IFN‐γ + RA, primary mast cells showed significantly increased expression of HLA‐DR (IFN‐γ versus NC, not significant; IFN‐γ + RA versus NC, *p* < 0.001; IFN‐γ + RA versus IFN‐γ, *p* < 0.001) and CD40 (IFN‐γ versus NC, *p* < 0.001; IFN‐γ + RA versus NC, *p* < 0.0001; IFN‐γ + RA versus IFN‐γ, *p* < 0.001) Consistent with the HMC‐1 results, CD80 and CD86 were also significantly upregulated following IFN‐γ + RA treatment (CD80, IFN‐γ versus NC, not significant; IFN‐γ + RA versus NC, *p* < 0.001; IFN‐γ + RA versus IFN‐γ, *p* < 0.001; CD86, IFN‐γ versus NC, not significant; IFN‐γ + RA versus NC, *p* < 0.0001; IFN‐γ + RA versus IFN‐γ, *p* < 0.001) (Figure S8B, Supporting Information). In addition, antigen uptake capacity (measured by OVA‐PE) and CCR7 expression were both significantly enhanced in IFN‐γ + RA–treated primary mast cells (OVA‐PE, IFN‐γ versus NC, *p* < 0.01; IFN‐γ + RA versus NC, *p* < 0.01; IFN‐γ + RA versus IFN‐γ, *p* < 0.01; CCR7, IFN‐γ versus NC, *p* < 0.001; IFN‐γ + RA versus NC, *p* < 0.0001; IFN‐γ + RA versus IFN‐γ, *p* < 0.001) (Figure S8C,D, Supporting Information).

### 3.6 Proinflammatory Mast Cells Regulate T Cell Recruitment and Activation

Given that CCR7 may be involved in mast cell homing, we further examined the spatial relationship between proinflammatory mast cells and TLS using mIHC. Our analysis revealed that proinflammatory mast cells were located in close proximity to TLS, suggesting their potential association with TLS formation or function (Figure S9A, Supporting Information). Cytokine profiles of HMC‐1 cells treated with IFN‐γ alone versus IFN‐γ + RA revealed the upregulation of proinflammatory cytokines like GM‐CSF, IFN‐γ, IL‐1α, IL‐1β, IL‐15, and chemokines like CCL1, CXCL9, and CXCL12 in the RA treatment group (Figure S9B,C, Supporting Information). These results suggest that RA treatment not only enhances the antigen‐presenting capabilities of mast cells but also bolsters adaptive immunity by promoting immune cell recruitment and activation.

Given that RA treatment induces mast cells to produce proinflammatory chemokines and cytokines, we further investigated the impact of these mediators on T cells. Transwell assay demonstrated that HMC‐1 cells treated with IFN‐γ + RA significantly attracted more T cells, both CD8^+^ and CD4^+^ T cells (CD3, IFN‐γ versus NC, *p* < 0.0001, IFN‐γ + RA versus NC, *p* < 0.0001, IFN‐γ + RA versus IFN‐γ, *p* = 0.0079; CD8, IFN‐γ versus NC, *p* < 0.0001, IFN‐γ + RA versus NC, *p* < 0.0001, IFN‐γ + RA versus IFN‐γ, *p* = 0.0079; CD4, IFN‐γ versus NC, *p* = 0.0022, IFN‐γ + RA versus NC, *p* = 0.0022, IFN‐γ + RA versus IFN‐γ, *p* < 0.0001) (Figure S9D, Supporting Information). Based on our CellphoneDB analysis (Figure S3H, Supporting Information), mast cells were predicted to interact with T cells via the CXCL16–CXCR6 axis. To functionally assess the role of this pathway in T cell recruitment, we performed a Transwell chemotaxis assay using increasing concentrations of recombinant CXCL16 in the lower chamber. We observed a dose‐dependent increase in T cell migration toward CXCL16 (Figure S9E, Supporting Information). Moreover, treatment with the CXCR6 antagonist ML339 in the upper chamber significantly impaired T cell migration (Figure S9F, Supporting Information), confirming that CXCL16–CXCR6 signaling is required for effective T cell chemotaxis. Flow cytometry showed that T cells cultured in conditioned medium from HMC‐1 cells treated with IFN‐γ + RA significantly upregulated TNF‐α expression (CD8, IFN‐γ versus NC, *p* = 0.0067, IFN‐γ + RA versus NC, *p* < 0.0001, IFN‐γ + RA versus IFN‐γ, *p* = 0.0037; CD4, IFN‐γ versus NC, *p* = 0.0022, IFN‐γ + RA versus NC, *p* = 0.0022, IFN‐γ + RA versus IFN‐γ, *p* < 0.0001) (Figure S9G, Supporting Information). These results indicated that RA‐treated HMC‐1 cells enhance the capacity of mast cells to activate T cells.

**Figure 5:**
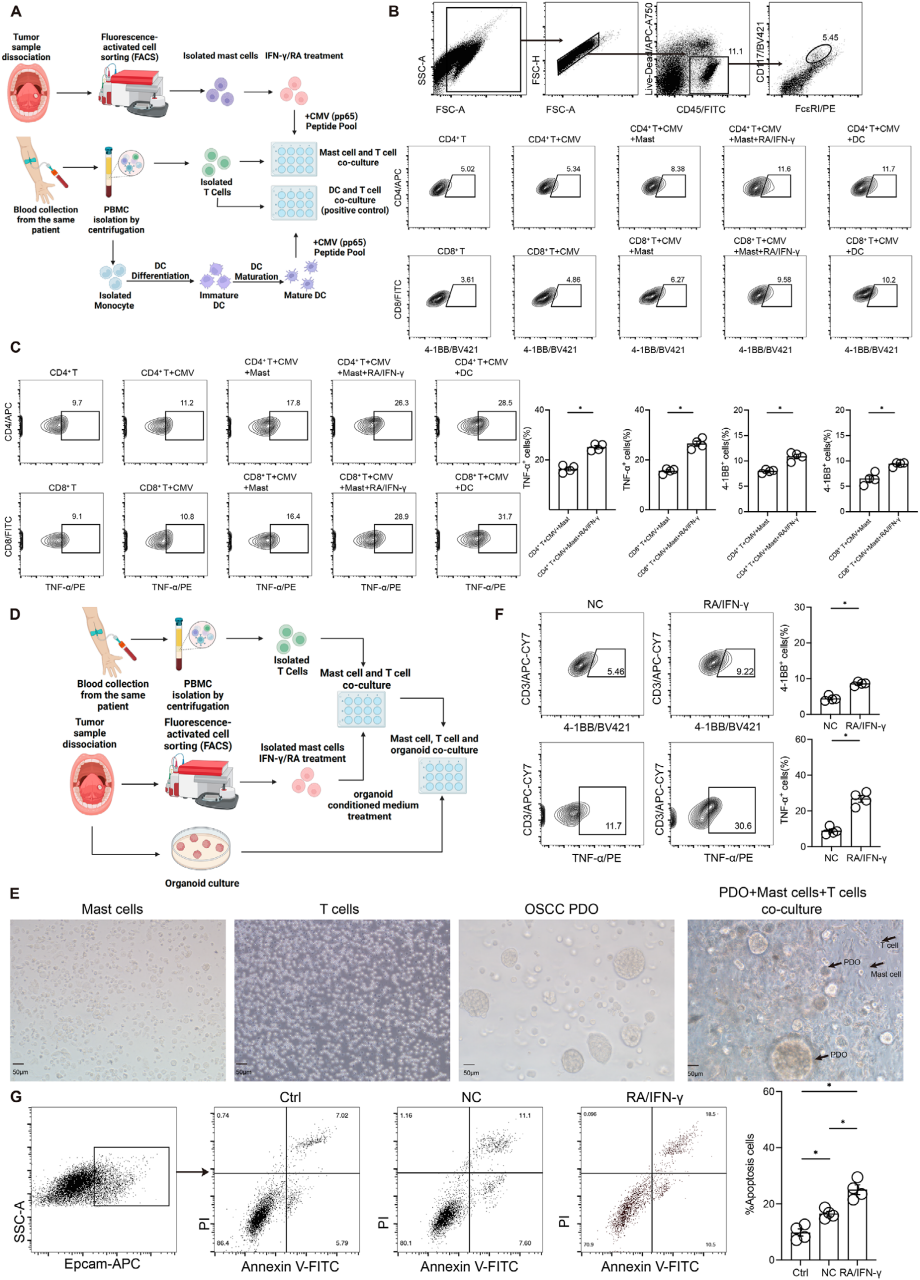
Proinflammatory mast cells regulate T cell recruitment and activation. A) Schematic illustration of the autologous mast cell and T cell antigen presentation assay (created with BioRender.com). B) Schematic diagram of the mast cell sorting process. C) Flow cytometry analysis showing CMV antigen presentation by mast cells to CD4⁺ and CD8⁺ T cells under different treatments, with T cell activation assessed by 4‐1BB and TNF‐α expression. D) Schematic illustration of the autologous mast cell, T cell, and patient‐derived organoid (PDO) co‐culture assay (created with BioRender.com). E) Representative image of mast cells, T cells, PDO, and mast cell, T cell, and PDO co‐culture. F) Flow cytometry analysis showing T cell activation in mast cell, T cell, and PDO co‐culture, assessed by 4‐1BB and TNF‐α expression. G) Flow cytometry analysis showing tumor cell killing in mast cell, T cell, and PDO co‐culture, assessed by Annexin V expression.

To directly assess the effect of RA on antigen presentation, we designed a mast cell‐T cell co‐culture system to evaluate the antigen‐presenting capacity of mast cells. Mast cells and T cells were isolated from the same patient to ensure MHC restriction, allowing for a direct assessment of antigen presentation. Mast cells were either left untreated or pre‐treated with IFN‐γ and RA before undergoing antigen loading with the CMV pp65 peptide pool. Co‐cultured T cell activation was measured by flow cytometry, using 4‐1BB and TNF expression as activation markers. A DC‐T cell co‐culture was included as a positive control for antigen presentation (**Figure** **5**A).

As shown in Figure 5B, mast cells were isolated from tumor tissues using CD45, CD117, and FCεRI as sorting markers. Flow cytometry analysis demonstrated that RA + IFN‐γ pre‐treated mast cells exhibited significantly enhanced activation of both CD4⁺ and CD8⁺ T cells, as indicated by increased 4‐1BB and TNF‐α expression compared to untreated or IFN‐γ‐treated mast cells. These results suggest that RA + IFN‐γ‐treated mast cells can directly present antigens to T cells and effectively activate them (Figure 5C).

To evaluate the ability of mast cells to mediate T cell cytotoxicity, we established a mast cell‐T cell‐tumor organoid co‐culture system. Tumor organoids were generated from freshly dissociated tumor tissues and expanded in Matrigel‐based 3D culture. Mast cells and T cells, both derived from the same patient, were co‐cultured following IFN‐γ and RA treatment of mast cells and subsequent exposure to tumor organoid‐conditioned medium. After co‐culture, tumor organoids were incorporated into the mast cell‐T cell suspension and embedded in Matrigel for 72 h. Following incubation, the co‐culture mixtures were dissociated and analyzed by flow cytometry. T cell activation was assessed by measuring 4‐1BB and TNF expression, while EpCAM‐positive tumor cells were evaluated using an Annexin V‐FITC/PI apoptosis assay to determine mast cell‐mediated T cell cytotoxicity (Figure 5D).

Representative image of sorted mast cells, T cells, PDO, and mast cell, T cell, and PDO co‐culture was shown in Figure 5E. Flow cytometry analysis demonstrated that T cells remained effectively activated in the RA + IFN‐γ‐treated mast cell co‐culture system, as evidenced by the upregulation of 4‐1BB and TNF‐α expression (Figure 5F). Figure 5G revealed a significant increase in apoptotic tumor cells in the RA + IFN‐γ‐treated mast cell co‐culture system, as indicated by Annexin V‐FITC/PI staining. These results suggest that RA + IFN‐γ‐treated mast cells enhance T cell‐mediated tumor cell killing, highlighting their potential role in promoting an effective anti‐tumor immune response.

Proinflammatory mast cells can activate T cells through several mechanisms. We analyzed the spatial location of proinflammatory mast cells and CXCL13, the marker of antigen‐specific T cells.^[^ ^52^ ^]^ As shown in Figure S10A (Supporting Information), we performed T cell clustering within the dataset, identifying distinct CD8_CXCL13 and CD4_CXCL13 subpopulations. By mapping the single‐cell dataset onto the spatial transcriptomic dataset, we observed colocalization of proinflammatory mast cells with CD8_CXCL13 (blue box) as well as CD4_CXCL13 (blue box), indicating their potential spatial interaction within the tissue microenvironment. Our mIHC analysis also indicated the proximity of proinflammatory mast cells and CD39^+^CXCL13^+^CD4^+^ T cells and CD39^+^CXCL13^+^CD8^+^ T cells (Figure S10B, Supporting Information). These results suggested that proinflammatory mast cells may activate T cells through direct cell‐cell interactions.

### 3.7 RA–RARα Signaling Induces CIITA‐Dependent Antigen‐Presenting Programming in Mast Cells

**Figure 6:**
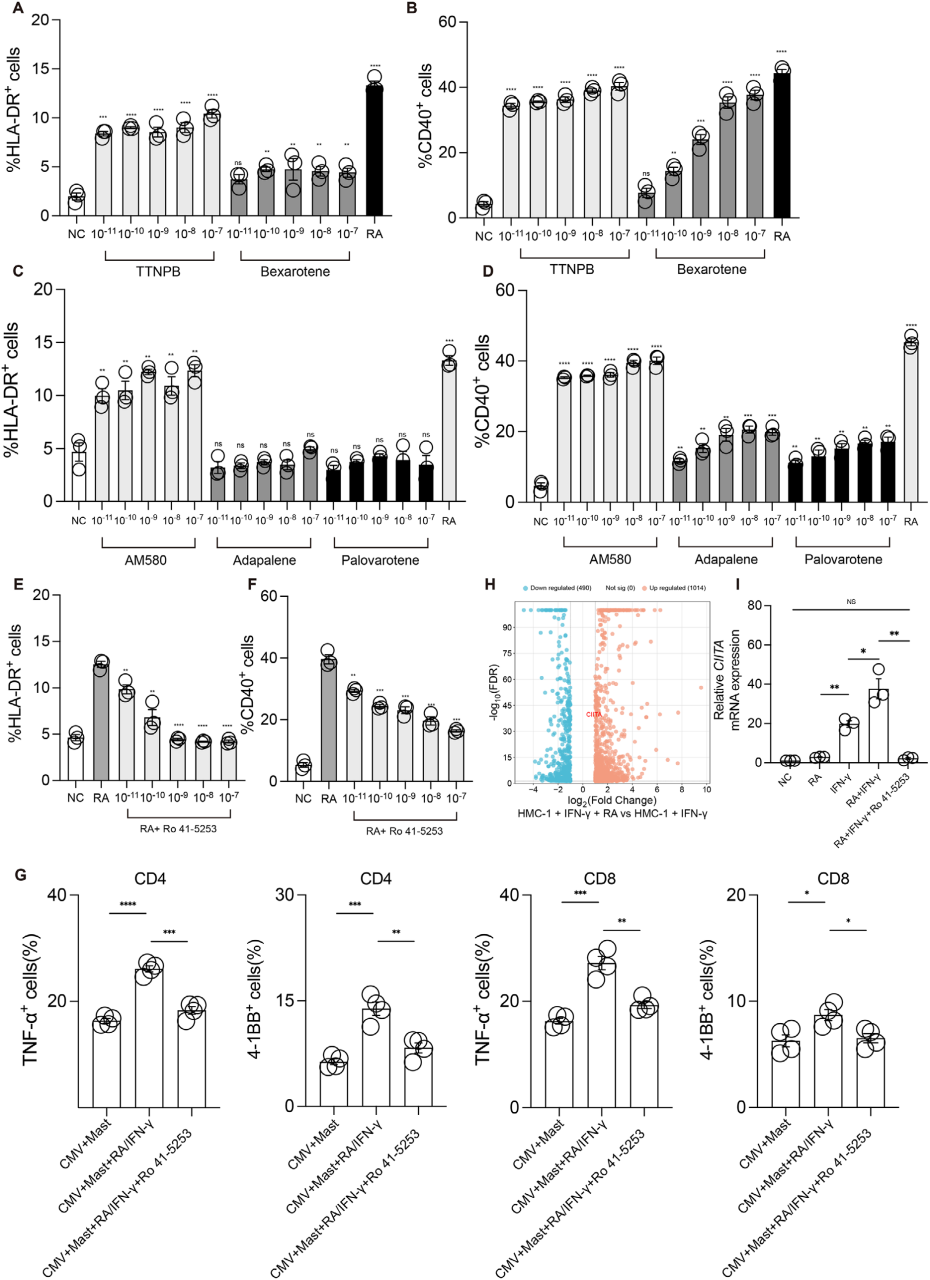
RA–RARα signaling induces CIITA‐dependent antigen‐presenting programming in mast cells. A) Flow cytometry analysis showing HLA‐DR expression after IFN‐γ +TTNPB and IFN‐γ +Bexarotene treatment for 48 h (*n* = 3). B) Flow cytometry analysis showing CD40 expression after IFN‐γ +TTNPB and IFN‐γ +Bexarotene treatment for 48 h (*n* = 3). C) Flow cytometry analysis showing HLA‐DR expression after IFN‐γ +AM580, IFN‐γ +Adapalene, and IFN‐γ +Palovarotene treatment for 48 h (*n* = 3). D) Flow cytometry analysis showing CD40 expression after IFN‐γ +AM580, IFN‐γ +Adapalene, and IFN‐γ +Palovarotene treatment for 48 h (*n* = 3). E) Flow cytometry analysis showing HLA‐DR expression after IFN‐γ +RA, IFN‐γ + RA+ Ro 41‐5253 treatment for 48 h (*n* = 3). F) Flow cytometry analysis showing CD40 expression after IFN‐γ +RA, IFN‐γ + RA+ Ro 41‐5253 treatment for 48 h (*n* = 3). G) Flow cytometry analysis showing CMV antigen presentation by mast cells to CD4⁺ and CD8⁺ T cells under different treatments, with T cell activation assessed by 4‐1BB and TNF‐α expression. H) Volcano plot showing significantly differentially expressed genes between the IFN‐γ + RA treatment group and the IFN‐γ treatment alone group. I) The mRNA expression of CIITA after IFN‐γ, RA, IFN‐γ +RA, IFN‐γ + RA+ Ro 41‐5253 treatment for 48 h (*n* = 3).

Building on the observed RA‐induced upregulation of HLA‐DR and CD40 (Figure 4D), we next investigated the underlying signaling pathway responsible for this effect. Pharmacologic interrogation of the RA signaling axis revealed that the pan‐RAR agonist TTNPB significantly enhanced HLA‐DR and CD40 expression at concentrations as low as 10^−11^ M, whereas the RXR agonist Bexarotene was ineffective at low concentrations and only showed partial activity at higher doses, likely due to off‐target RAR activation (**Figure** **6**A,B). These findings indicate that RAR, rather than RXR, is the primary mediator of RA‐induced mast cell activation, prompting us to further dissect the contribution of individual RAR isoforms.

To identify the specific RAR isoform involved, we applied isoform‐selective agonists and found that the RARα agonist AM580 elicited the strongest upregulation of HLA‐DR and CD40, while RARβ (Adapalene) and RARγ (Palovarotene) agonists had weaker effects (Figure 6C,D). Notably, co‐treatment with the RARα antagonist Ro 41‐5253 abrogated RA‐induced upregulation of both markers (Figure 6E,F), confirming the central role of RARα.

Functionally, RA + IFN‐γ–treated mast cells significantly enhanced T cell activation in an autologous co‐culture system, whereas RARα blockade impaired this response (Figure 6G). RNA‐seq analysis further revealed that RA robustly upregulated CIITA, the master regulator of MHC class II gene expression (Figure 6H). This was confirmed by qPCR, which showed that Ro 41‐5253 significantly reduced CIITA expression to levels below those induced by IFN‐γ alone (Figure 6I). These results suggest that RA–RARα signaling not only amplifies the IFN‐γ response but also serves as an essential driver of CIITA‐dependent transcriptional programming in mast cells. Together, these findings establish RARα as the key mediator of RA‐induced mast cell reprogramming and identify CIITA as a critical downstream effector driving the acquisition of antigen‐presenting features.

### 3.8 Validation of Proinflammatory Mast Cells in Immunotherapy‐Related Single‐Cell and Bulk Transcriptomic Datasets

**Figure 7:**
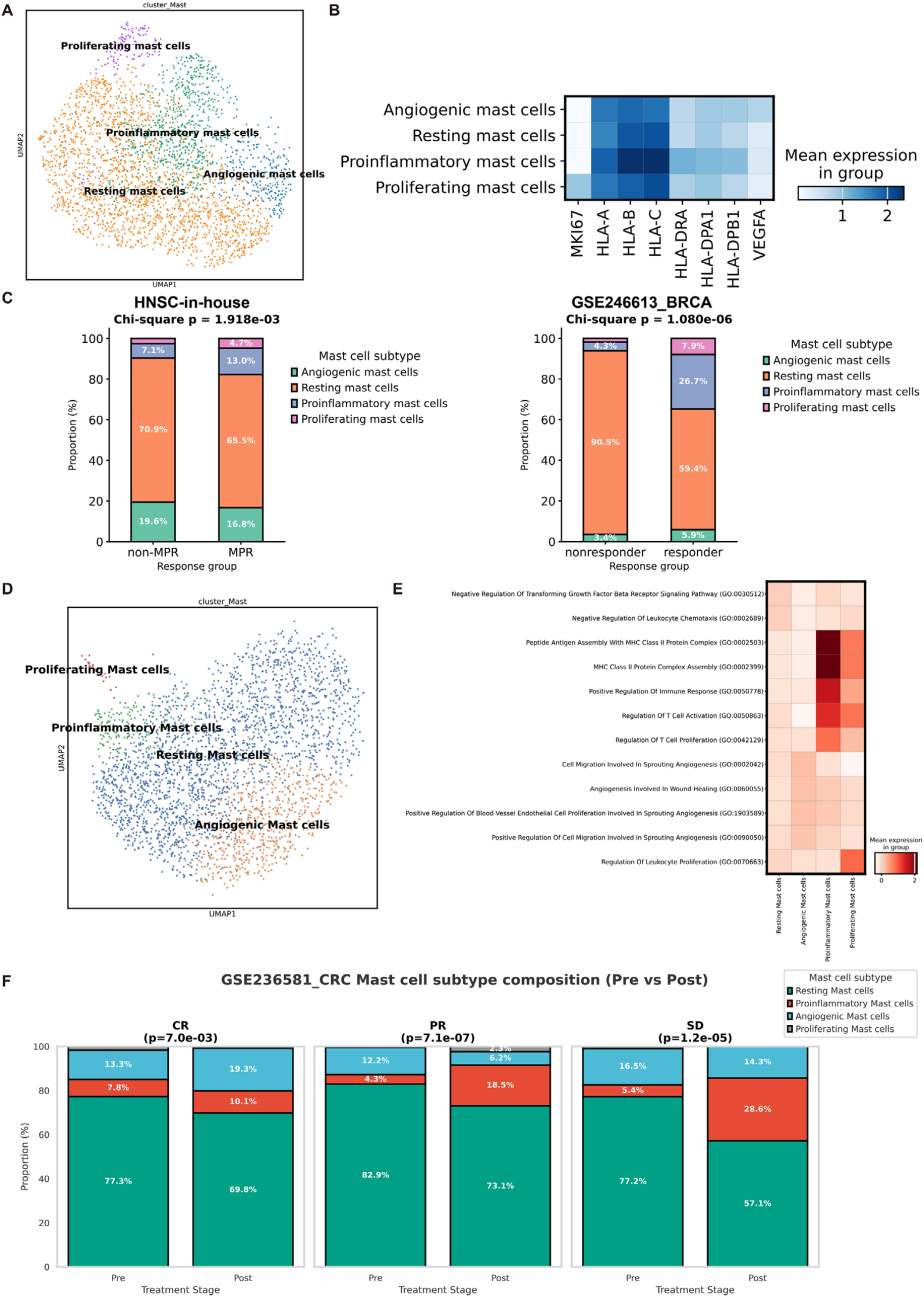
Validation of proinflammatory mast cells in immunotherapy‐related single‐cell datasets. A) UMAP visualization of the four mast subclusters in the validation cohort. B) Matrix plot displaying marker genes for the four mast subclusters. C) Stacked bar graphs depicting the proportions of the mast cell subclusters between responders and non‐responders. D) UMAP visualization of the four mast subclusters in GSE236581. E) Matrix plot displaying marker pathways enriched in the four mast subclusters. F) Stacked bar graphs depicting the proportions of the mast cell subclusters across different treatment stages and clinical response groups (CR, PR, SD).

To further validate our findings in the context of immune checkpoint blockade, we analyzed two independent single‐cell datasets: our in‐house cohort of eight HNSC patients who received neoadjuvant immunochemotherapy^[^ ^53^ ^]^ and GSE246613 (BRCA).^[^ ^54^ ^]^ Together, these datasets contained 2724 mast cells for integrative analysis (**Figure** **7**A,B). Across both cohorts, pretreatment biopsies from responders exhibited higher proportions of proinflammatory mast cells than those from non‐responders (Figure 7C), suggesting that proinflammatory mast cell enrichment prior to ICB therapy may indicate a more immunologically active microenvironment.

Consistent with these findings, analysis of ICB‐related bulk RNA‐seq cohorts from the Cancer Immunology Data Engine (CIDE)^[^ ^55^ ^]^ demonstrated that tumors with high PMC‐signature (TPSAB1, HLA‐DRA, HLA‐DPA1, HLA‐DPB1, HLA‐DRB1, CD74) expression exhibited superior survival outcomes across multiple cancer types (Figure S11A,B, Supporting Information), reinforcing the link between PMC polarization and effective antitumor immunity.

To further characterize the dynamic changes of mast cell states during ICB therapy, we analyzed the single‐cell dataset GSE236581,^[^ ^40^ ^]^ which includes paired tumor samples collected before and after ICB treatment. Reclustering of mast cells revealed four subtypes—resting, proinflammatory, angiogenic, and proliferating (Figure 7D)—validated by pathway enrichment (Figure 7E). Quantification of mast cell composition across different treatment stages and clinical response groups (CR, PR, SD) revealed a consistent trend: PMC proportions increased following ICB treatment across all response groups (Figure 7F). Although total mast cell numbers were limited, this trend suggests therapy‐induced activation and polarization toward an immune‐stimulatory phenotype. Collectively, these findings indicate that mast cell polarization dynamics mirror the evolving immune status of the tumor microenvironment during ICB therapy, supporting the role of proinflammatory mast cells as a potential biomarker and functional amplifier of antitumor immunity.

Additionally, spatial transcriptomic profiling using digital spatial profiling (DSP) was performed on tumor samples from six HNSC patients, with 12 regions of interest (ROIs) analyzed (Figure S11C, Supporting Information). As shown in Figure S11D (Supporting Information), the proinflammatory mast cell score was significantly elevated in regions from MPR samples (*p* = 0.0252). Furthermore, the proinflammatory mast cell score was significantly correlated with both the exhausted CD8 T cell score (*p* = 0.0008) and the cytotoxic CD4 T cell score (*p* < 0.0001), suggesting that proinflammatory mast cells may colocalize with antigen‐specific T cells (Figure S11E, Supporting Information). These findings suggest that proinflammatory mast cells are enriched in HNSC patients with a better response to neoadjuvant immunochemotherapy and may play a role in activating antigen‐specific T cells.

**Figure 8:**
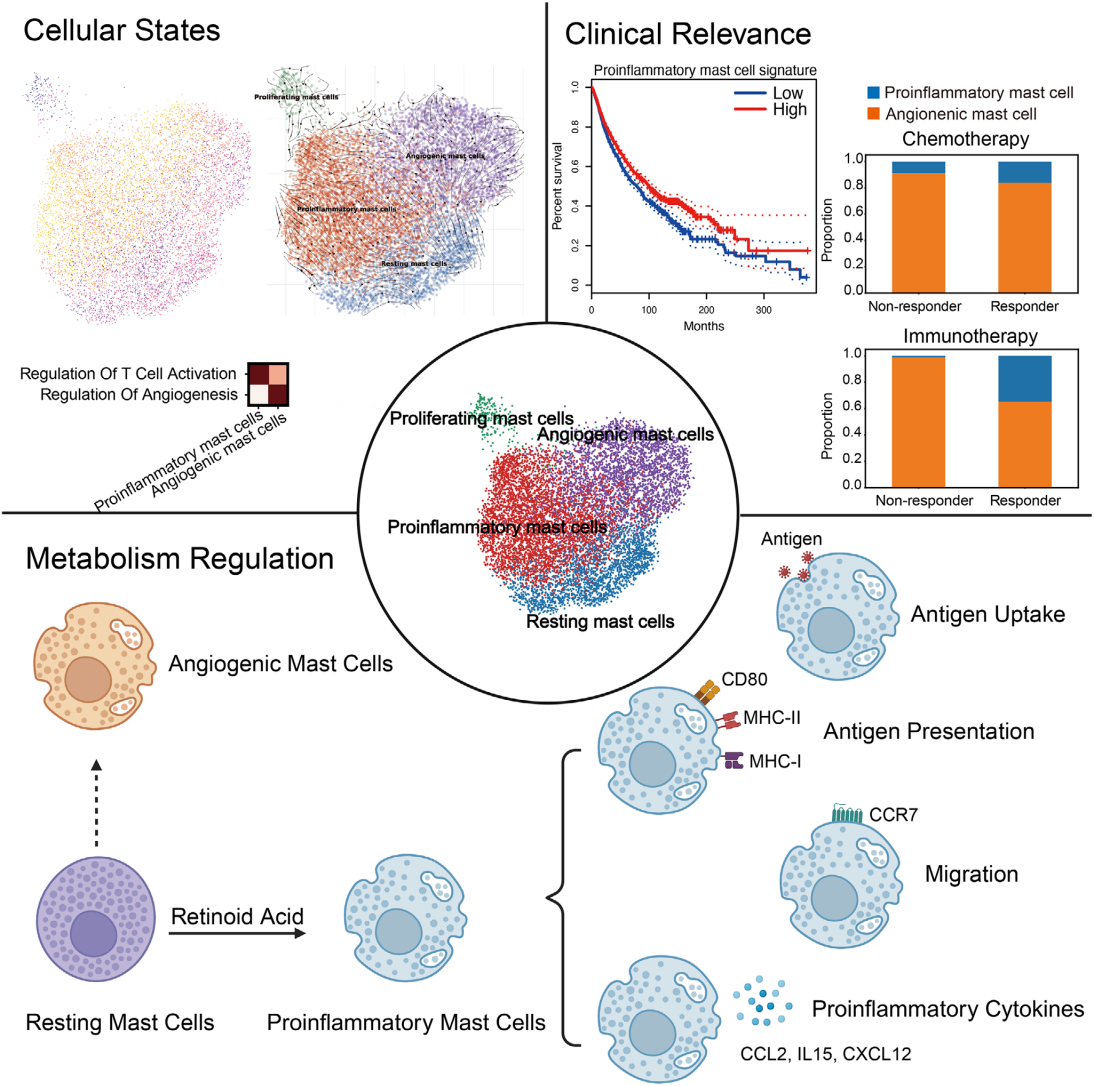
Schematic diagram. Schematic summarizing the main findings of the study. Proinflammatory mast cells are a subcluster of mast cells that highly express MHC‐II molecules and produce proinflammatory chemokines and cytokines. Proinflammatory mast cell infiltration is associated with better survival and improved response to immunotherapy and chemotherapy. RA is capable of inducing proinflammatory mast cell polarization. Proinflammatory mast cells mediate adaptive anti‐tumor immune responses through several mechanisms, including antigen uptake and presentation, migration to lymphoid organs or lymphoid structures, and T cell recruitment and activation via chemokine and cytokine production (created with BioRender.com).

In summary, we found proinflammatory mast cells are a subcluster of mast cells that highly express MHC‐II molecules and produce proinflammatory chemokines and cytokines. Proinflammatory mast cell infiltration is associated with better survival and improved response to immunotherapy and chemotherapy. RA is capable of inducing proinflammatory mast cell polarization. Proinflammatory mast cells mediate adaptive anti‐tumor immune responses through several mechanisms, including antigen uptake and presentation, migration to lymphoid organs or lymphoid structures, and T cell recruitment and activation via chemokine and cytokine production (**Figure** **8**).

## 4 Discussion

Recently, mast cells have been categorized into pro‐tumorigenic and anti‐tumorigenic subtypes based on their roles in cancer.^[^ ^1^ ^]^ In this study, we comprehensively characterized the transcriptional and functional heterogeneity of mast cells by integrating large‐scale single‐cell datasets. Our most significant finding is the identification of proinflammatory mast cells, which exhibit antigen‐presenting and proinflammatory properties across multiple cancers. Importantly, these cells can be stimulated by RA, ultimately exerting a protective effect in cancer patients. By providing new insights into mast cell heterogeneity and their role in cancer, this study advances our understanding of the complex interplay between mast cells and T cells and underscores the potential of targeting mast cells for cancer therapy.

Mast cells are a non‐negligible population within the tumor microenvironment, despite their small numbers. Their role in cancer has been a topic of debate. Consistent with previous findings,^[^ ^11^ ^]^ we observed higher mast cell infiltration in tumor tissues compared to uninvolved normal tissues in most cancer types. Notably, mast cell infiltration was higher in normal colorectal tissues than in CRC, mirroring results from previous studies.^[^ ^56^ ^]^ However, these studies reported opposing prognostic implications of mast cell infiltration in CRC, highlighting the heterogeneity of mast cells. Similarly, we found that normal cervix tissues exhibited higher mast cell infiltration than CESC, which aligns with previous research.^[^ ^57^ ^]^ Similarly, Cheng et al.^[^ ^11^ ^]^ also reported increased mast cell presence in the adjacent normal uterus, suggesting a role for mast cells in reproductive processes.^[^ ^58^ ^]^ In our study, mast cell infiltration was lower in metastatic tumor tissues, although recent research showed that choroid plexus mast cells drive tumor‐associated hydrocephalus,^[^ ^59^ ^]^ indicating that this rare population of mast cells may play a significant role in metastatic sites as well.

Moreover, our results indicated that mast cells interact closely with immune cells through CXCL16‐CXCR6, TNF‐TNFRSF1B, IFNG‐Type‐II‐IFNR, and SEMA4D‐CD72 interactions. Mast cells can secrete CXCL16 to attract CXCR6^+^ T cells, a marker for resident memory T cells that plays a role in immunosurveillance and immunotherapy.^[^ ^60^ ^]^ Additionally, mast cells produce TNF‐α, which promotes T cell survival and activation.^[^ ^61^ ^]^ In turn, T cell‐secreted IFN‐γ can induce the expression of MHC class II and co‐stimulatory molecules on mast cells, granting them the ability to process and present antigens directly to T cells.^[^ ^62^, ^63^ ^]^ Moreover, the SEMA4D‐CD72 interaction between mast cells and B cells can promote B cell activation, proliferation, and antibody production.^[^ ^64^ ^]^ Importantly, mast cells can induce B cells to produce IgE, which can mediate mast cell degranulation.^[^ ^65^ ^]^ These results indicated that mast cells may play an important role in regulating adaptive anti‐tumor immunity in the tumor microenvironment.

Regarding immunotherapy, recent studies have demonstrated that mast cell infiltration is associated with unfavorable outcomes in anti‐PD‐1 therapy, which is consistent with our findings. Somasundaram et al.^[^ ^66^ ^]^ found that mast cells were enriched in anti‐PD‐1 non‐responders and that depletion of mast cells improved anti‐PD‐1 treatment outcomes in a humanized mouse melanoma model. Similarly, Li et al.^[^ ^46^ ^]^ reported that cromolyn sodium enhanced the efficacy of PD‐1 immunotherapy, suggesting that inhibiting mast cell degranulation and cytokine release benefits anti‐PD‐1 therapy. Another study using ketotifen to inhibit mast cell activation also improved the efficacy of anti‐PD‐L1 therapy in sarcoma.^[^ ^67^ ^]^ Our results indicate that proinflammatory mast cell infiltration is associated with better responses to anti‐PD‐1 therapy, emphasizing the critical role of proinflammatory mast cells in immunotherapy and suggesting new strategies for mast cell targeting, from depletion and stabilization to proinflammatory mast cell polarization. Johansson et al.^[^ ^68^ ^]^ reported that peritumoral mast cells stimulate peritumoral angiogenesis and prostate tumor growth. Liu et al.^[^ ^69^ ^]^ found that tumor stroma‐infiltrating mast cells were associated with worse survival and higher recurrence after adjuvant chemotherapy. Consistent with these observations, our study found that angiogenic mast cells, which express high levels of VEGFA, were located near blood vessels and associated with poor outcomes. Conversely, Hempel et al.^[^ ^70^ ^]^ reported intratumoral mast cells may protect against prostate cancer recurrence, serving as a prognostic biomarker after prostatectomy. Bodduluri et al.^[^ ^5^ ^]^ found that intratumoral and peritumoral mast cell‐derived LTB_4_ is essential for CD8^+^ T cell recruitment and effective antitumor immunity. Our findings align with these studies, showing that proinflammatory mast cells, which express MHC‐II molecules, are located at the tumor‐stroma interface and are associated with better survival. We hypothesize that proinflammatory mast cells capture tumor antigens at the tumor‐stroma interface and mediate adaptive antitumor immunity in situ.

Cheng et al.^[^ ^11^ ^]^ stratified mast cells on the basis of VEGFA and TNF expression and argued that a high TNF/VEGFA ratio underpins anti‐tumor activity. Their results show that a pronounced TNF^+^ dominance occurs almost exclusively in nasopharyngeal carcinoma (NPC), whereas most other cancers exhibit a VEGFA^+^‐dominant profile. By contrast, our pan‐cancer analysis did not reveal a discrete TNF^+^ subset; instead, we uncovered a proinflammatory mast cell population distinguished by high MHC‐II gene expression while TNF levels remain comparable to other states and, importantly, are stably represented across multiple cancer entities (Figure 2G). Very recently, Wu et al.^[^ ^71^ ^]^ reported that ∼30 % of intra‐tumoral mast cells in triple‐negative breast cancer (TNBC) are CD74^+^ antigen‐presenting mast cells and are indispensable for effective tumor control, a result that aligns with our observation that the protective mast‐cell program is characterized by high MHC‐II rather than elevated TNF. The fact that TNF^+^ mast cells peak only in NPC—a cancer with strong Epstein–Barr‐virus‐driven inflammation—suggests that viral etiology may amplify TNF signaling,^[^ ^72^, ^73^ ^]^ whereas the MHC‐II‐defined program we report is relevant to a broader spectrum of solid tumors. Taken together, these observations reinforce the view that the antigen‐presentation capacity of mast cells—rather than TNF release—plays a central, though likely not exclusive, role in shaping anti‐tumor immunity and improving responses to immunotherapy.

To mechanistically explain the observed heterogeneity of mast cells across tumor types, particularly the emergence of the proinflammatory subset associated with improved prognosis and immunotherapy response, we next explored potential regulatory signals that may drive their polarization. RA, a metabolite of vitamin A known for its immunomodulatory effects, was identified through metabolic pathway enrichment as a candidate inducer of this mast cell phenotype. This prompted us to investigate whether RA could functionally reprogram mast cells into an antigen‐presenting, proinflammatory state, thus linking our single‐cell discovery with a mechanistic and therapeutic framework.

RA is widely recognized for its dual roles in inducing differentiation and inhibiting proliferation in various cellular contexts. Its use in treating acute promyelocytic leukemia (APL) represents a paradigm shift, being the first example of a targeted anti‐tumor agent demonstrating therapeutic efficacy.^[^ ^74^ ^]^ Within the tumor microenvironment, RA has been shown to increase MHC‐I expression on tumor cells, thereby enhancing their visibility to the immune system,^[^ ^75^ ^]^ and to promote the survival of tumor‐specific CD8^+^ T cells,^[^ ^76^ ^]^ bolstering anti‐tumor immunity. Regarding myeloid cells, Rao et al. reported that RA promotes inflammatory macrophage activity, which synergizes with radiation therapy to enhance anti‐tumor responses.^[^ ^77^ ^]^ Additionally, Nefedova et al. demonstrated that RA induces the differentiation of myeloid‐derived suppressor cells (MDSCs), thereby reducing their immunosuppressive activity and enhancing T‐cell‐mediated anti‐tumor responses.^[^ ^78^ ^]^ This differentiation also alleviates tumor hypoxia, normalizes tumor vasculature, and improves the efficacy of antiangiogenic therapies.^[^ ^79^ ^]^ Clinical studies further underscore RA's therapeutic potential. A phase II trial showed that combining RA with chemotherapy improved survival in advanced non‐small‐cell lung cancer (NSCLC) patients, with an acceptable toxicity profile.^[^ ^80^ ^]^ Moreover, a phase Ib/II trial combining RA with pembrolizumab effectively reduced circulating MDSCs and achieved a 71% overall response rate in metastatic melanoma patients.^[^ ^81^ ^]^

In our study, RA treatment was shown to induce proinflammatory mast cell polarization. These cells expressed high levels of MHC‐II molecules, chemokines, and cytokines. Importantly, previous studies have demonstrated that RA treatment induces iNOS^+^ TNF‐α^+^ inflammatory macrophages, which enhance T cell activation, priming, and infiltration. Moreover, IFN‐γ has been shown to synergistically enhance the induction of inflammatory macrophages by RA.^[^ ^77^ ^]^ These findings align with our results, suggesting that RA and its associated pathways play a central role in myeloid cell development. Previous studies have reported that IFN‐γ can induce MHC‐II expression on mast cells, enabling them to present antigens to CD4^+^ T cells.^[^ ^62^ ^]^ Additionally, Stelekati et al.^[^ ^82^ ^]^ have reported that mast cells can present antigen to CD8^+^ T cells, indicating their potential role as antigen‐presenting cells within the tumor microenvironment, thereby regulating T cell activation. Furthermore, our findings showed that proinflammatory mast cells tend to reside near CD39^+^CXCL13^+^ T cells, which are known to be tumor‐reactive T cells that recognize tumor antigens and play a pivotal role in immune checkpoint blockade. Taken together, these observations suggest that inducing proinflammatory mast cell polarization through RA treatment could be an effective approach to activating T cells.

Moreover, we found that RA treatment enhances mast cell production of inflammatory chemokines and cytokines, such as CXCL12 and IL‐15. CXCL12 has also been shown to stimulate T cell chemotaxis and activation,^[^ ^83^ ^]^ while IL‐15 is known to promote the generation, proliferation, and activity of anti‐tumor NK cells and CD8^+^ T cells, without promoting Tregs.^[^ ^84^ ^]^ Collectively, these results indicate that RA treatment not only enhances mast cell antigen presentation capabilities but also bolsters their ability to activate T cells through the production of inflammatory chemokines and cytokines.

Furthermore, our study revealed that RA treatment significantly upregulated CCR7 expression in mast cells, potentially enabling CCR7‐expressing mast cells to migrate to tertiary lymphoid structures enriched with CCR7 ligands. Rivellese et al.^[^ ^85^ ^]^ reported that mast cells residing in TLS promote B cell activation and antibody production in rheumatoid arthritis. In summary, we propose that inducing proinflammatory mast cells facilitates the formation of TLS in cancer.

Mechanistically, we identified CIITA—a master regulator of MHC class II gene expression—as a downstream target of RA–RARα signaling in mast cells. Pharmacological blockade of RARα abolished RA‐induced upregulation of CIITA, HLA‐DR, and CD40, indicating that RA orchestrates antigen presentation machinery through transcriptional control. This regulatory axis is consistent with previous findings in dendritic cells, where deletion of Rara impairs maturation, antigen uptake, and processing, ultimately diminishing their ability to activate T cells.^[^ ^86^ ^]^ Our results now extend this paradigm to mast cells, which have historically been overlooked in the context of antigen presentation. Importantly, we also found that RA treatment—but not IFN‐γ alone—significantly upregulated co‐stimulatory molecules CD80 and CD86 on mast cells. This is notable because multiple studies have reported that IFN‐γ fails to induce CD80/CD86 expression in mast cells,^[^ ^51^, ^62^, ^63^ ^]^ suggesting that RA is essential for conferring the full spectrum of antigen‐presenting capabilities, including both MHC‐II and co‐stimulatory signals. Moreover, our identification of CIITA as a direct downstream target of RA–RARα signaling is further supported by prior work in multiple myeloma cells, where RARα activation was shown to induce CIITA expression and restore MHC‐II expression.^[^ ^87^ ^]^ Together, these findings establish a coherent mechanistic framework in which RA–RARα signaling induces antigen‐presenting programming in mast cells by transcriptionally activating CIITA and promoting the expression of both MHC‐II components and co‐stimulatory molecules.

RA‐driven MHC‐II up‐regulation on mast cells presupposes an IFN‐γ “first signal.” Although baseline IFN‐γ varies widely across—and even within—tumor types, several on‐therapy biopsy studies indicate that contemporary chemo‐immunotherapy frequently induces an early IFN‐γ surge. In melanoma, nivolumab given alone or with ipilimumab increased intratumoral IFN‐γ transcripts within 2–4 weeks, independent of radiographic outcome.^[^ ^88^ ^]^ In colorectal cancer, Yost et al.^[^ ^89^ ^]^ observed that neoadjuvant PD‐1 blockade led to a robust increase in IFN‐γ score within 4 weeks in both mismatch‐repair‐deficient and ‐proficient tumors. Neoadjuvant chemo‐immunotherapy in non‐small‐cell lung cancer likewise increased intratumoral IFN‐γ after combined chemotherapy and PD‐1 blockade, with the change most evident in lesions in non‐MPR group.^[^ ^90^ ^]^ These findings suggest that administering RA during the early phase of chemo‐immunotherapy—when an IFN‐γ peak is most probable—should minimize concerns about insufficient IFN‐γ. If IFN‐γ remains low, brief priming measures such as low‐dose radiotherapy^[^ ^91^ ^]^ or emerging STING agonist^[^ ^92^ ^]^ can raise it to a permissive level and may create a more favorable window for RA. Hence, IFN‐γ variability is chiefly a timing issue rather than an absolute barrier, and RA can still function as a practical amplifier of mast‐cell antigen presentation within an appropriately primed microenvironment.

RA treatment is generally well tolerated; its common adverse events—fatigue, headache, low‐grade fever, dermatitis, hypertriglyceridaemia, and mild gastrointestinal upset—are usually self‐limiting. Serious complications such as differentiation syndrome, pseudotumour cerebri, myocarditis, myositis, Sweet's syndrome, and ulceration are uncommon but well‐documented.^[^ ^93^ ^]^ These toxicities are acceptable during the relatively brief courses used to treat APL, yet they could restrict longer administration schedules envisaged for solid tumors. Several measures may mitigate this risk. First, RAR‐α‐selective agonists such as tamibarotene (Am80) achieve mast‐cell activation at lower concentrations and have shown a more favorable safety profile in clinical trials.^[^ ^94^ ^]^ Second, local or nano‐encapsulated delivery systems—liposomal RA or polymeric nanoparticles—can concentrate the drug within the tumor while reducing systemic exposure.^[^ ^95^ ^]^ Third, because IFN‐γ acts as a permissive “first signal,” RA might be required only during the early treatment window, allowing for short, intermittent dosing schedules. Finally, ex vivo priming of autologous mast cells with RA before reinfusion could, in principle, circumvent systemic exposure altogether. Although each approach warrants further pharmacokinetic and safety evaluation, current data indicate that RA toxicity is a manageable hurdle rather than a fundamental barrier to clinical translation.

Our study has certain limitations. First, the cohorts analyzed involve the combination of PD‐1 blockade with conventional chemotherapy, which makes it difficult to specifically attribute the predictive power of mast cells to PD‐1 blockade alone. The observed associations may be influenced by chemotherapy, immunotherapy, or their combined effects. Future studies focusing on cohorts treated exclusively with PD‐1 blockade are needed to validate the specific role of mast cells in predicting PD‐1 blockade efficacy.

Second, a limitation of this study is the absence of in vivo validation experiments to assess the effect of RA on mast cell polarization and anti‐tumor immunity within a living organism. Although our in vitro and ex vivo assays—using primary mast cells and patient‐derived tumor models—provided strong functional evidence, in vivo studies will be important in future work to further confirm these findings and evaluate therapeutic relevance in a more complex physiological context.

Third, a key limitation of this study lies in the relatively small number of mast cells captured across single‐cell datasets, which inherently limits the statistical power for subgroup comparisons and cross‐cohort validation. The rarity of mast cells within the tumor microenvironment makes it challenging to obtain sufficiently large samples for robust statistical modeling. Additionally, differences in sequencing depth, tissue sampling, and data preprocessing among studies may introduce variability that affects the precision of mast cell subtype quantification. Future work will benefit from the inclusion of larger‐scale, multi‐center single‐cell and spatial transcriptomic datasets—particularly from immunotherapy‐treated patients—to further validate and refine the associations between mast cell polarization, retinoic acid signaling, and antitumor immunity.

This study presents a comprehensive analysis of mast cell heterogeneity across multiple cancer types, revealing the critical role of proinflammatory mast cells in modulating the tumor microenvironment. By integrating single‐cell RNA sequencing and spatial transcriptomics, we identified distinct mast cell subtypes and demonstrated that proinflammatory mast cells, characterized by high expression of MHC‐II molecules, are associated with improved survival outcomes and enhanced responses to immunotherapy. Additionally, we discovered that RA can induce proinflammatory mast cell polarization, further augmenting their antigen‐presenting capabilities and promoting T cell activation. These findings underscore the potential of targeting mast cell polarization as a novel therapeutic strategy to enhance the efficacy of cancer immunotherapy, paving the way for improved treatment outcomes in cancer patients.

## 5 Conclusion

In this study, we generated a comprehensive pan‐cancer single‐cell and spatial transcriptomic atlas of human mast cells across ten tumor types, uncovering pronounced transcriptional and spatial heterogeneity. We discovered a proinflammatory mast cell subset—characterized by elevated MHC‐II and chemokine expression—that is preferentially enriched in tumors responsive to immune checkpoint inhibitors. This association was validated by multiplex immunohistochemistry in independent cohorts of head and neck squamous cell carcinoma, colorectal cancer, and lung cancer patients, where higher proinflammatory mast cell density correlated with better pathological response and survival. Mechanistically, we demonstrate that RA polarizes mast cells toward this antigen‐presenting, proinflammatory state, enhancing CD4⁺ and CD8⁺ T cell recruitment and activation within the tumor microenvironment. Together, these findings reveal a protective immunoregulatory role for proinflammatory mast cells in cancer immunotherapy and highlight mast cell polarization as a novel target to further improve ICI efficacy.

## Conflict of Interest

The authors declare no conflict of interest.

## Author Contributions

L.Z.Z., S.Q.R., Y.H.L., R.X.C., and L.Q. contributed equally to this work, and Z.J.H., H.T.C., Q.X.L., J.S.L., and T.J.L. jointly supervised this work. **Lizao Zhang**: Conceptualization, resources, data curation, formal analysis, supervision, validation, investigation, visualization, methodology, writing–original draft, writing–review and editing. **Siqi Ren**: Data curation, software, formal analysis, validation, investigation, visualization, methodology, writing–original draft, writing–review and editing. **Yuhui Li**: Data curation, formal analysis, investigation, visualization, methodology, writing–original draft. **Rongxi Chen**: Data curation, software, formal analysis, validation, investigation, visualization, methodology, writing–original draft. **Ling Qiu**: Data curation, software, formal analysis, validation, investigation, visualization, methodology, writing–original draft. **Yongmei Tan**: Data curation, software, formal analysis, validation, investigation, visualization, methodology, writing–original draft. **Suling Chen**: Resources, data curation, validation. **Huiqian Wu**: Software, formal analysis, visualization. **Lianxi Mai**: Data curation. **Xiao Tan**: Data curation, validation, methodology. **Xin Liu**: Investigation, methodology. **Peisheng Liang**: Investigation, methodology. **Shijia Kuang**: Resources, data curation. **Liansheng Wang**: Data curation. **Jingkang Liu**: Investigation, methodology. **Jintao Li**: Validation, investigation, methodology. **Yanyan Li**: Data curation. **Qiuping Xu**: Software. **Yongzhi Su**: Validation, investigation. **Yuehai Luo**: Data curation, supervision. **Binyan Wu**: Validation, investigation. **Zijing Huang**: Conceptualization, data curation, funding acquisition, writing–review and editing. **Haotian Cao**: Conceptualization, supervision, funding acquisition, writing–review and editing. **Qunxing Li**: Conceptualization, supervision, funding acquisition, writing–review and editing. **Jinsong Li**: Conceptualization, resources, supervision, funding acquisition, writing–original draft, writing–review and editing. **Tianjun Lan**: Conceptualization, data curation, software, formal analysis, supervision, funding acquisition, validation, investigation, visualization, methodology, writing–original draft, writing–review and editing.

## Supporting information



Supporting Information

Supporting Information

Supporting Information

## Data Availability

The data that support the findings of this study are available from the corresponding author upon reasonable request.
