# Supplementary material for: Retinoic Acid Reprograms Mast Cells Toward a Proinflammatory State to Enhance Antitumor Immunity
Source: Adv Sci (Weinh). 2025 Nov 27;13(6):e09340. doi: 10.1002/advs.202509340 (PMC12866796; doi:10.1002/advs.202509340)
Supplement: Supplementary file 1 — Supporting Information [file ADVS-13-e09340-s003.docx]

**Retinoic acid reprograms mast cells toward a proinflammatory state to enhance antitumor immunity**

**Supplemental Figure legends**

**Supplemental Figure 1: Characteristics of pan-cancer cohort**

**A.** Stacked bar graph showing the proportion of tissue types collected by cancer type. Pie chart showing the proportion of tissue types across all samples.

**B.** Stacked bar graph showing the proportion of treatment types collected by cancer type. Pie chart showing the proportion of treatment types across all samples.

**C.** **UMAP plots before (left) and after (right) batch correction**

**Supplemental Figure 2: UMAP visualization showing marker genes for the major cell clusters.**

**Supplemental Figure 3: Pan-cancer analysis of mast cells**

**A.** Boxplot showing the proportion of mast cells among immune cells in primary tumor tissues collected by cancer type. The figures on the bars indicate the proportion of mast cells.

**B.** Boxplot showing the proportion of mast cells among immune cells in uninvolved normal tissues collected by cancer type. The figures on the bars indicate the proportion of mast cells.

**C.** Boxplot showing the proportion of mast cells among immune cells in metastatic tumor tissues collected by cancer type and metastatic site. The figures on the bars indicate the proportion of mast cells.

**D.** Boxplot showing the proportion of mast cells among myeloid cells in primary tumor tissues collected by cancer type. The figures on the bars indicate the proportion of mast cells.

**E.** Boxplot showing the proportion of mast cells among myeloid cells in uninvolved normal tissues collected by cancer type. The figures on the bars indicate the proportion of mast cells.

**F.** Boxplot showing the proportion of mast cells among myeloid cells in metastatic tumor tissues collected by cancer type and metastatic site. The figures on the bars indicate the proportion of mast cells.

**G.** Scatter plot showing the correlation between mast cell infiltration and the infiltration of T cells, B cells, and plasma cells across various cancer types.

**H.** Dot plot displaying the predicted receptor-ligand interactions between mast cells and lymphoid cells, highlighting key interactions that may mediate immune responses.

**I.** Kaplan-Meier plots showing the prognostic impact of mast cell infiltration in specific cancer types, including glioblastoma multiforme (GBM), cervical squamous cell carcinoma and endocervical adenocarcinoma (CESC), pancreatic adenocarcinoma (PAAD), and kidney renal clear cell carcinoma (KIRC).

**J.** Stacked bar graphs comparing the proportion of mast cells among immune cells (left) and among myeloid cells (right) in primary tumor tissues between responders and non-responders to immunotherapy. The figures on the bars indicate the proportion of mast cells.

**Supplemental Figure 4: Pan-cancer analysis of mast cell subclusters**

**A.** Heatmap showing the pathways enriched in resting, activated, and proliferating mast cells, highlighting key biological processes associated with each mast cell state.

**B.** Stacked bar graph showing the proportion of activated mast cells among all mast cells in primary tumor tissues collected by cancer type. The figures on the bars indicate the proportion of activated mast cells.

**C.** Stacked bar graph showing the proportion of activated mast cells among all mast cells in uninvolved normal tissues across different cancer types. The figures on the bars indicate the proportion of activated mast cells.

**D.** Stacked bar graph showing the proportion of activated mast cells among all mast cells in metastatic tumor tissues across different cancer types. The figures on the bars indicate the proportion of activated mast cells.

**E.** Stacked bar graph showing the proportion of activated mast cells among all mast cells in primary tumor tissues between responders and non-responders to immunotherapy. The figures on the bars indicate the proportion of activated mast cells.

**F.** Stacked bar graph showing the proportion of proinflammatory mast cells among activated mast cells in uninvolved normal tissues across different cancer types. The figures on the bars indicate the proportion of proinflammatory mast cells.

**G.** Stacked bar graph showing the proportion of proinflammatory mast cells among activated mast cells in metastatic tumor tissues across different cancer types. The figures on the bars indicate the proportion of proinflammatory mast cells.

**H.** Stacked bar graph showing the proportion of proinflammatory mast cells among all mast cells in primary tumor tissues between responders and non-responders to immunotherapy. The figures on the bars indicate the proportion of proinflammatory mast cells.

**I.** Matrix plot showing the expression of PD1 among mast cell subclusters.

**Supplemental Figure 5: mIHC verifies the prognostic significance of mast cell subtypes in the CRC cohort**

Boxplots comparing mast cell density, proinflammatory mast cell density, proinflammatory mast cell proportion, angiogenic mast cell density, and angiogenic mast cell proportion between patients with long and short survival in the CRC cohort. Representative mIHC images from long- and short-survival cases are shown.

**Supplemental Figure 6: mIHC verifies the prognostic significance of mast cell subtypes.**

**A.** Kaplan-Meier survival curves for the HNSC cohort, stratified by the median expression levels of total mast cells, proinflammatory mast cells, and angiogenic mast cells. Survival differences between high and low groups were assessed.

**B.** Kaplan-Meier survival curves for the CRC cohort, stratified by the median expression levels of total mast cells, proinflammatory mast cells, and angiogenic mast cells. Survival differences between high and low groups were assessed.

**C.** Kaplan-Meier survival curves for the Lung cancer cohort, stratified by the median expression levels of total mast cells, proinflammatory mast cells, and angiogenic mast cells. Survival differences between high and low groups were assessed.

**D.** Representative image showing proinflammatory mast cells localized at the tumor-stromal border.

**E.** Representative image showing angiogenic mast cells located near CD34+ blood vessels.

**Supplemental Figure 7: Retinoic acid regulates proinflammatory mast cell polarization**

**A.** Flow cytometry analysis showing HLA-DQ and CD74 expression after RA, IFN-γ and IFN-γ + RA treatment for 48 hours (n = 6).

**B.** Volcano plot showing significantly differentially expressed genes between the IFN-γ + RA treatment group and the IFN-γ treatment alone group.

**C.** Dot plot showing the pathways enriched in the IFN-γ + RA treatment group.

**D.** Flow cytometry analysis showing CCR7 expression after RA, IFN-γ and IFN-γ + RA treatment for 48 hours (n = 6).

**Supplemental Figure 8: Retinoic acid regulates proinflammatory mast cell polarization in primary mast cells**

**A.** Schematic diagram of the mast cell sorting process.

**B.** Flow cytometry analysis showing HLA-DR, CD40, CD80 and CD86 expression on primary mast cells after RA, IFN-γ and IFN-γ + RA treatment for 48 hours (n = 3).

**C.** Flow cytometry analysis showing OVA-PE uptake by primary mast cells after RA, IFN-γ and IFN-γ + RA treatment for 48 hours (n = 3).

**D.** Flow cytometry analysis showing CCR7 expression on primary mast cells after RA, IFN-γ and IFN-γ + RA treatment for 48 hours (n = 3).

**Supplemental Figure 9: Proinflammatory mast cells recruit and activate T cells**

**A.** Representative mIHC images showing the spatial localization of proinflammatory mast cells near tertiary lymphoid structures (TLS), defined by CD3, CD20, and CD21 markers.

**B.** Original plots from the RayBio Human Cytokine Antibody Array, with color-coded boxes representing corresponding cytokines.

**C.** Bar plot displaying the fold change in cytokine expression between the IFN-γ + RA treatment group and the IFN-γ treatment alone group. The expression intensity was calculated after averaging and normalization by the intensity of the positive control.

**D.** Flow cytometry analysis showing the number of migrated CD3, CD4, and CD8 T cells in a Transwell assay after different treatments (n = 5).

**E.** Flow cytometry analysis showing the number of migrated CD3, CD4, and CD8 T cells in a Transwell assay after different treatments (n = 3).

**F.** Flow cytometry analysis showing the number of migrated CD3, CD4, and CD8 T cells in a Transwell assay after different treatments (n = 3).

**G.** Flow cytometry analysis showing TNF-α expression in T cells cultured with complete medium (NC), conditioned medium from HMC-1 cells treated with RA for 48 hours (RA), conditioned medium from HMC-1 cells treated with IFN-γ for 48 hours (IFN-γ), and conditioned medium from HMC-1 cells treated with IFN-γ + RA for 48 hours (IFN-γ + RA) (n = 6).

**Supplemental Figure 10: Proinflammatory mast cells colocalize with CXCL13+ T cells**

**A.** UMAP visualization of the 7 T cell subclusters. Dot plot displaying marker genes for the 7 T cell clusters. Spatial expression plots from HNSC samples. Blue box represents proinflammatory mast cells and CXCL13+CD8 T cells colocalization area, while red box represents proinflammatory mast cells and CXCL13+CD4 T cells colocalization area.

**B.** mIHC analysis showing colocalization of proinflammatory mast cells with CD39^+^CXCL13^+^CD4^+^ T cells and CD39^+^CXCL13^+^CD8^+^ T cells. White arrows indicate proinflammatory mast cells, white arrowheads indicate CD39^+^CXCL13^+^CD8^+^ T cells, and white triangles indicate CD39^+^CXCL13^+^CD4^+^ T cells.

**Supplemental Figure 11: Validation of proinflammatory mast cells in immunotherapy-related bulk RNA-seq datasets and in-house spatial transcriptomic dataset**

**A.** Box plots showing the association of the PMC signature and other ICB-related gene signatures with overall survival (OS) across multiple immune checkpoint blockade (ICB) cohorts. Higher expression of the PMC signature is correlated with better OS in most datasets.

**B.** Kaplan–Meier plots showing that high PMC signature expression is significantly associated with better survival in three ICB cohorts: Foy2022 NSCLC, Foy2022 Head & Neck, and Gide2019 Melanoma.

**C.** HE slides from 6 HNSC patients treated with neoadjuvant immunochemotherapy. White circles indicate the selected ROIs.

**D.** Boxplots comparing the proinflammatory mast cell scores between MPR and non-MPR ROIs.

**E.** Scatter plots illustrating the correlation between the proinflammatory mast cell score and the exhausted CD8 T cell score, as well as the correlation between the proinflammatory mast cell score and the cytotoxic CD4 T cell score.
