# Supplementary figures and images for: Retinoic Acid Reprograms Mast Cells Toward a Proinflammatory State to Enhance Antitumor Immunity
Source: Adv Sci (Weinh). 2025 Nov 27;13(6):e09340. doi: 10.1002/advs.202509340 (PMC12866796; doi:10.1002/advs.202509340)

Supplemental Figure 5

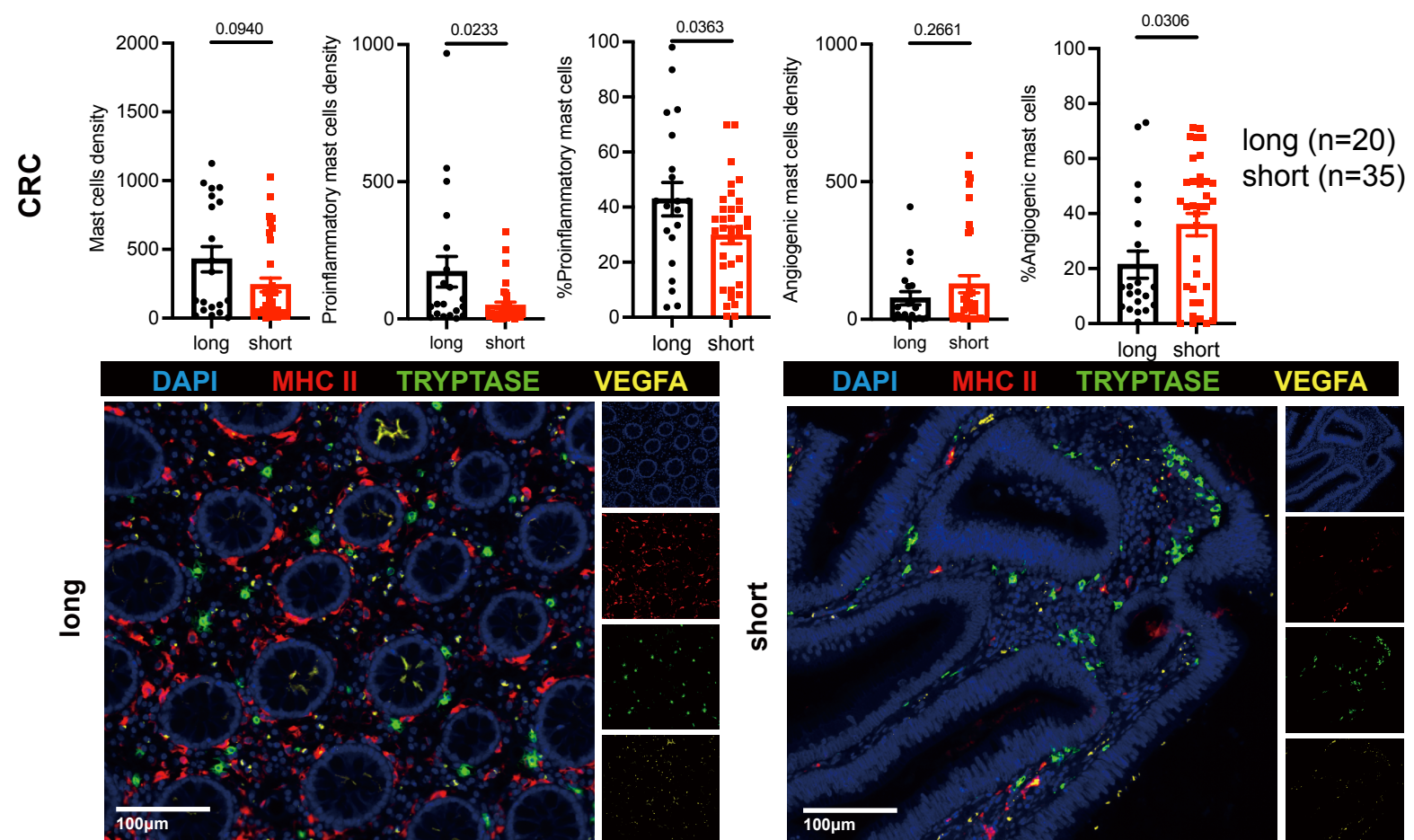

Supplement: Supplementary file 2 — Supporting Information [file ADVS-13-e09340-s001.zip › Mast_Sup5_revised.pdf]

Supplemental Figure 6

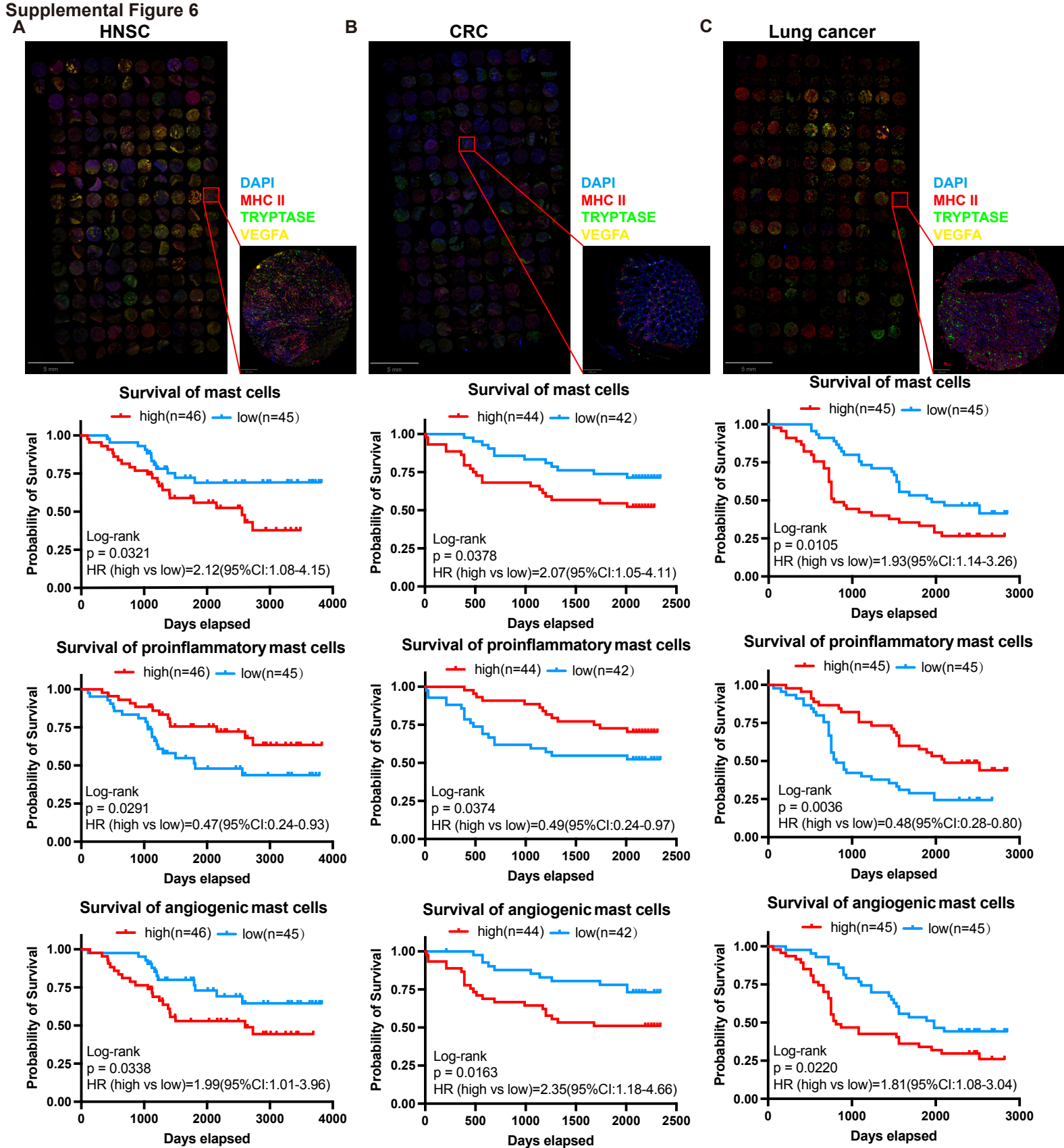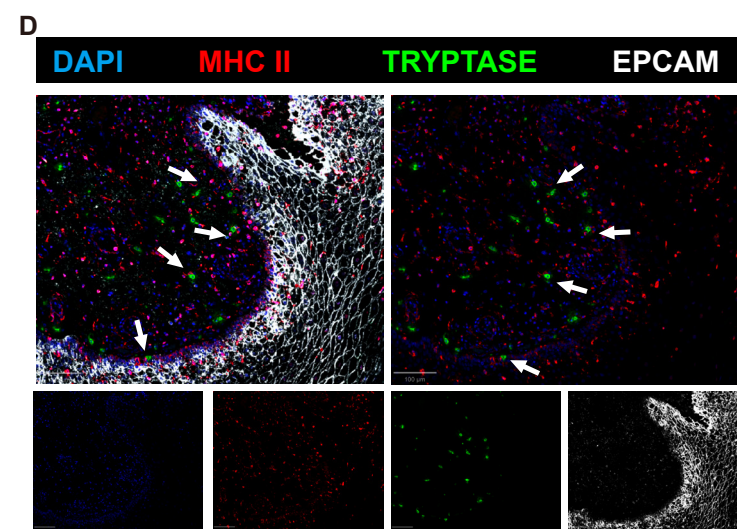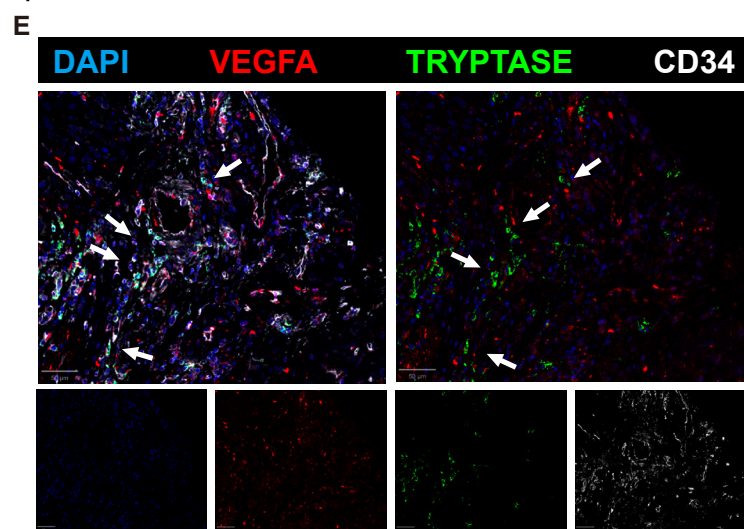

Supplement: Supplementary file 2 — Supporting Information [file ADVS-13-e09340-s001.zip › Mast_Sup6_revised.pdf]

Supplemental Figure 7

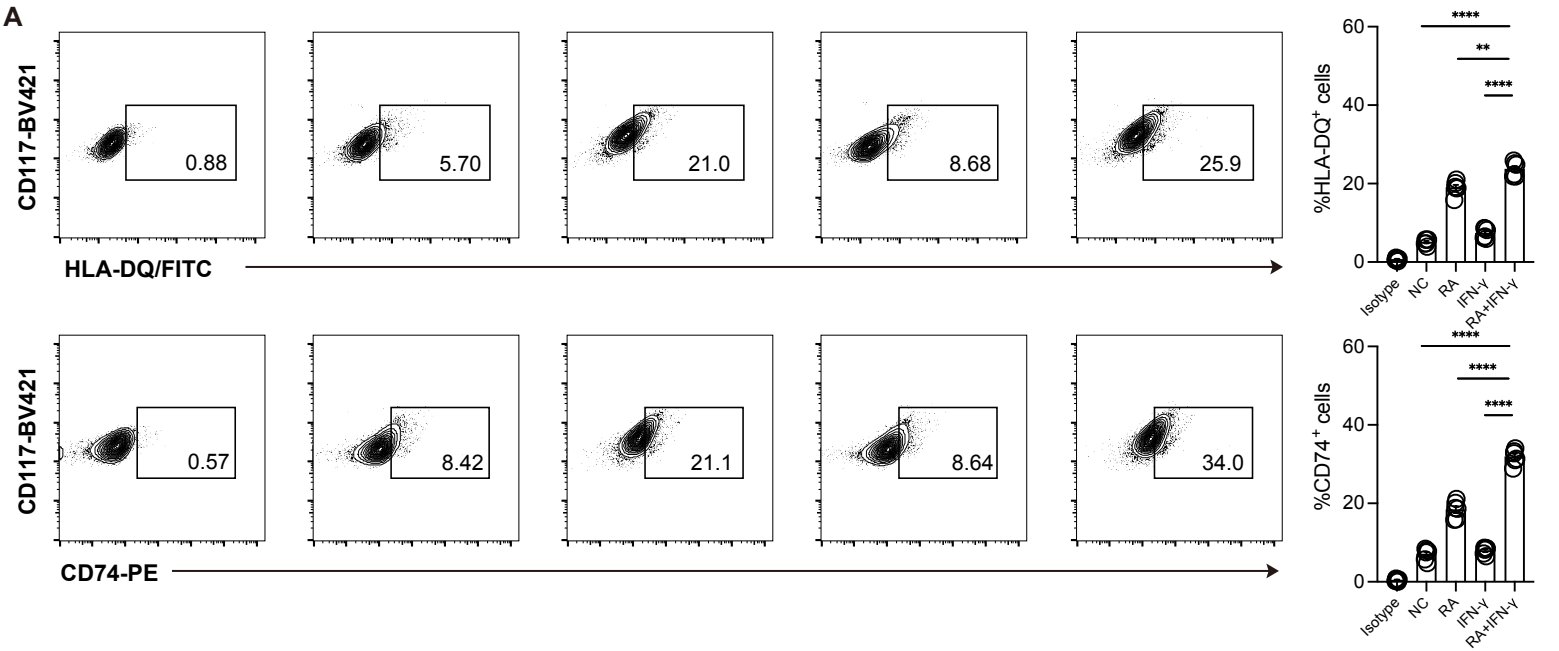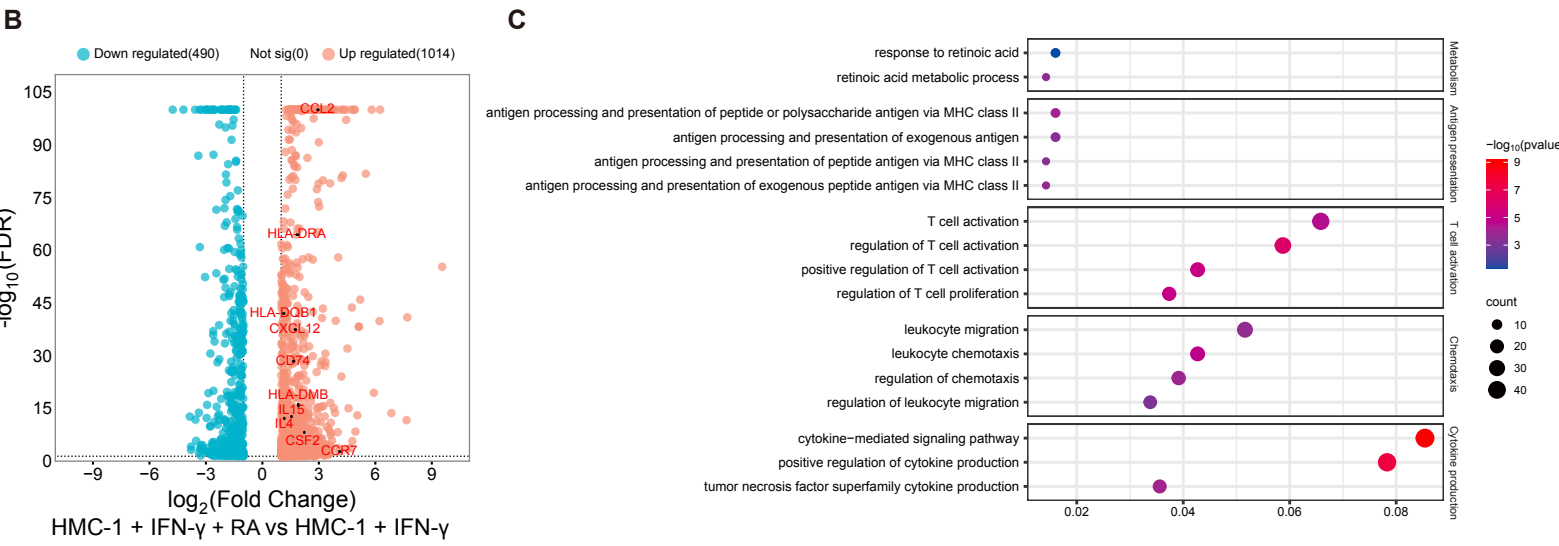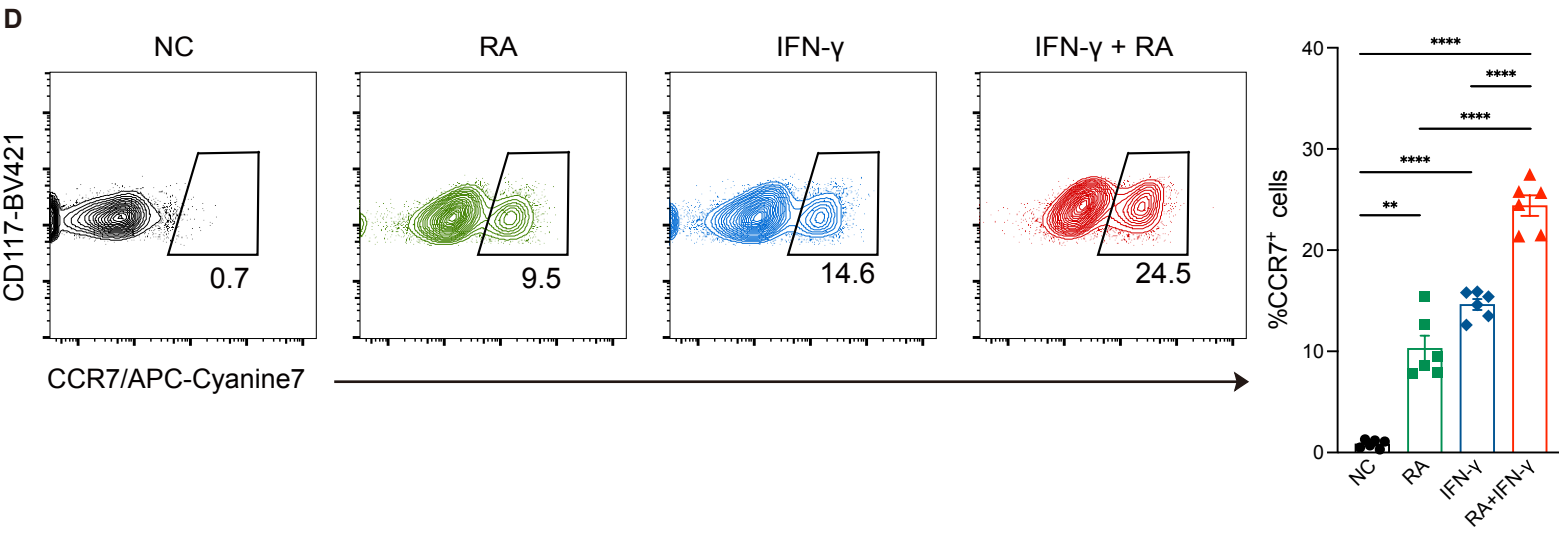

Supplement: Supplementary file 2 — Supporting Information [file ADVS-13-e09340-s001.zip › Mast_Sup7_revised.pdf]

A

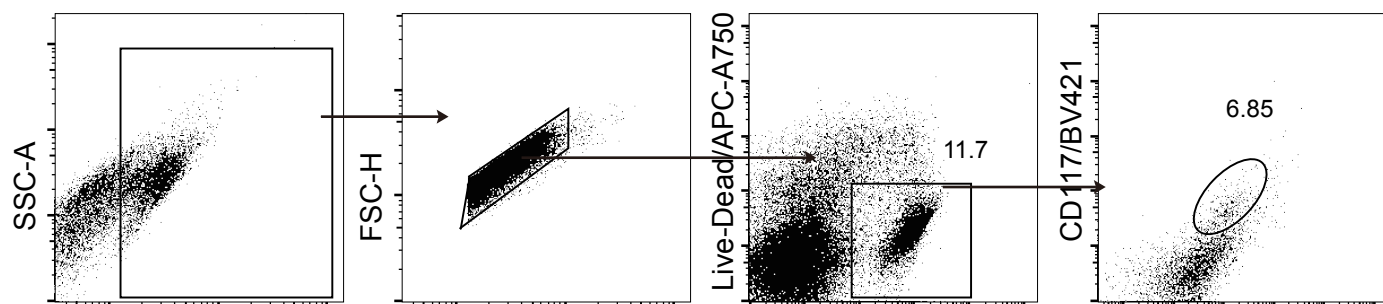

B

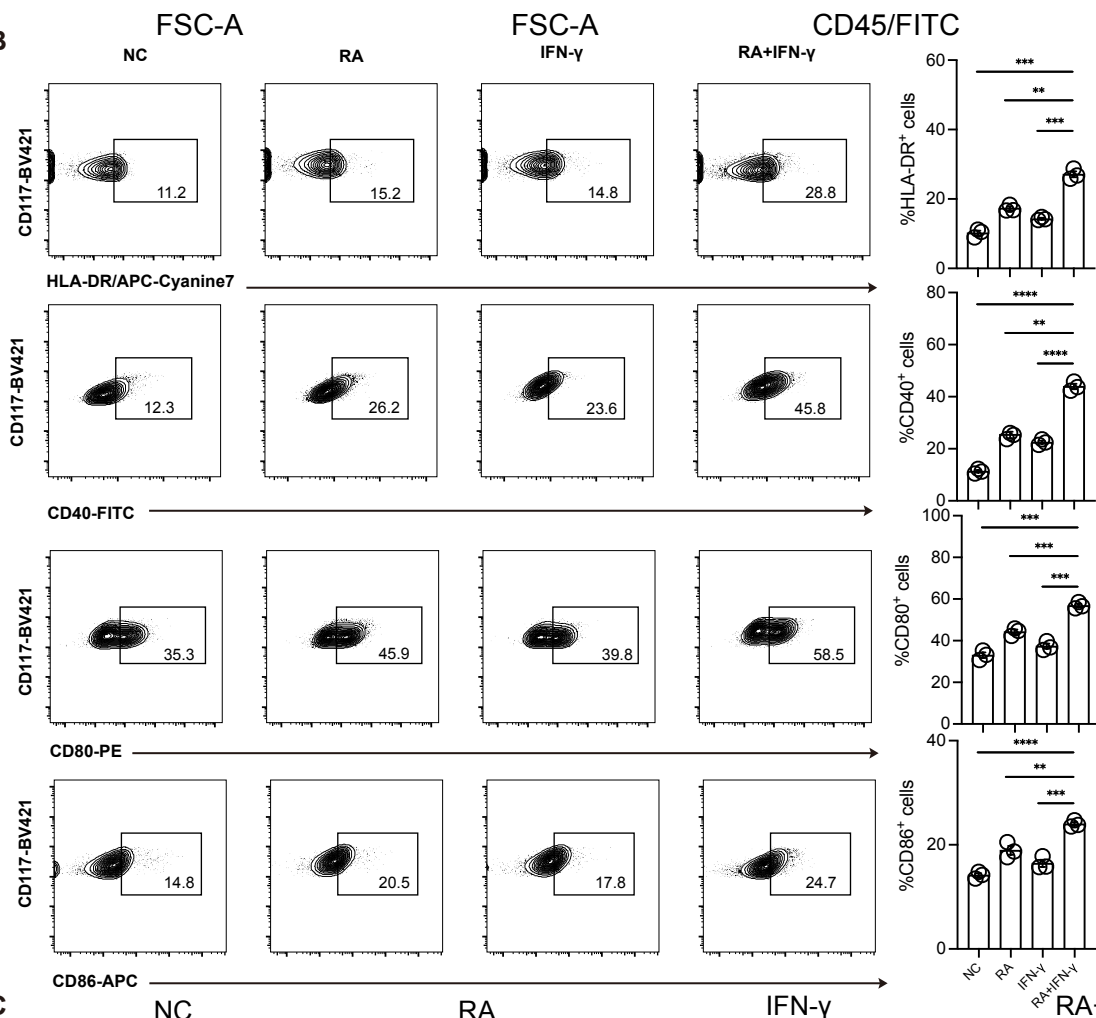

C

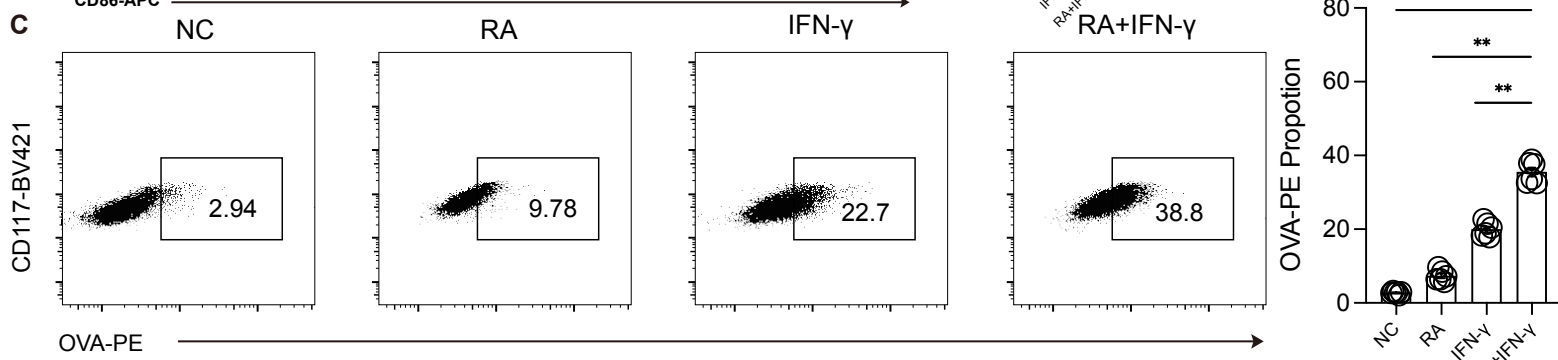

D

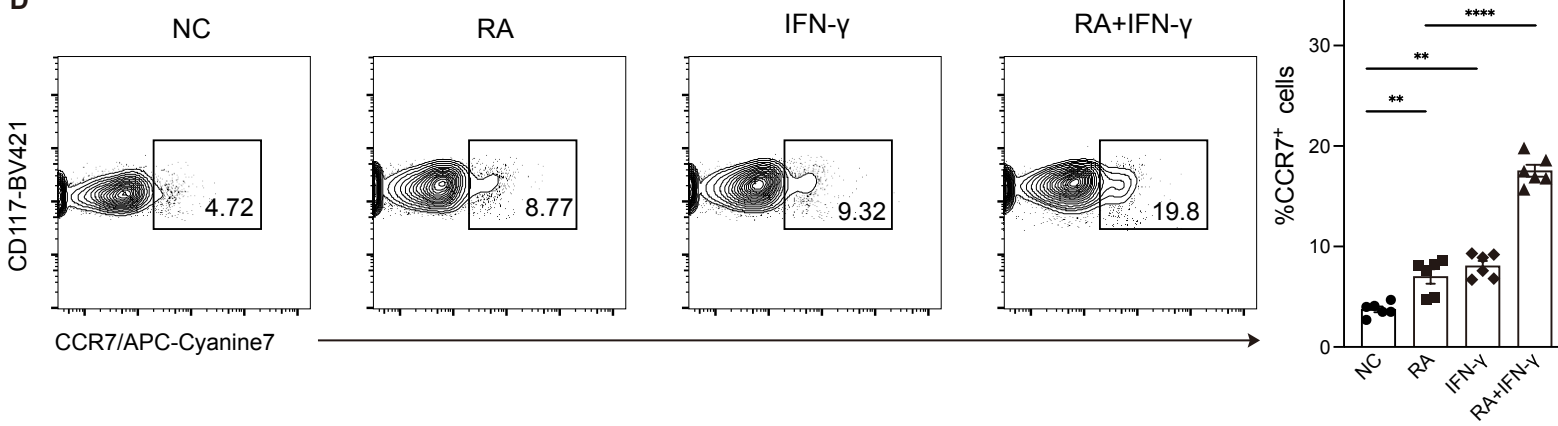

Supplement: Supplementary file 2 — Supporting Information [file ADVS-13-e09340-s001.zip › Mast_Sup8_revised.pdf]

Supplemental Figure 9

A

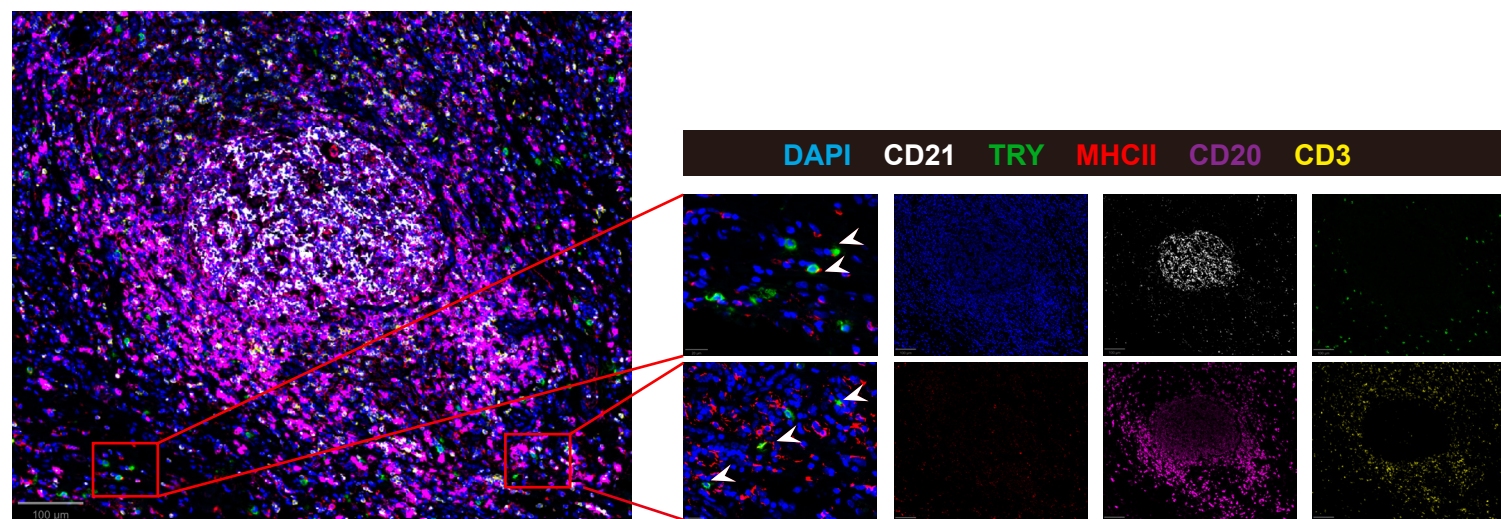

B

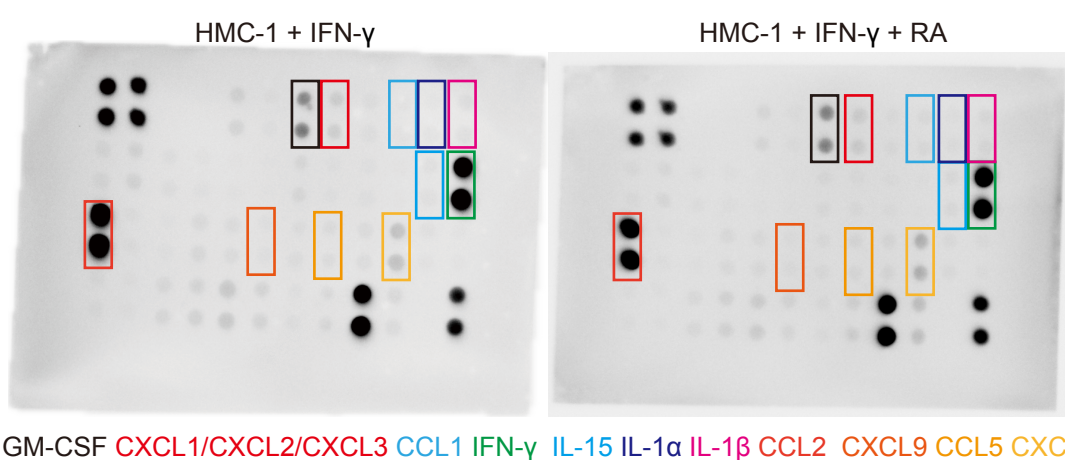

C

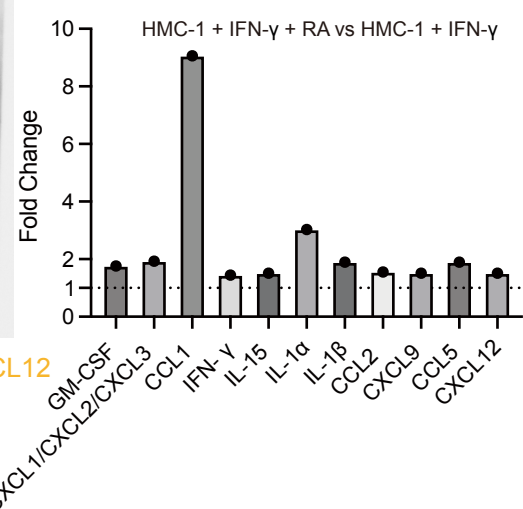

D

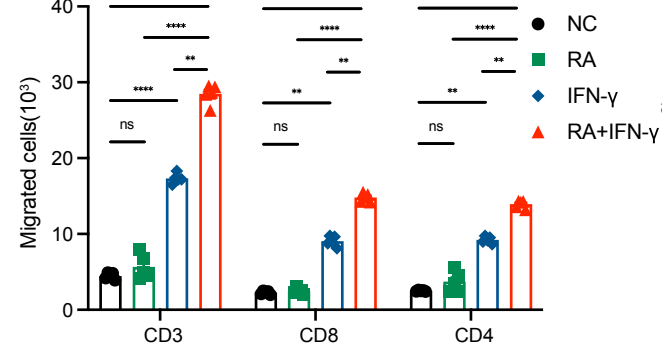

E

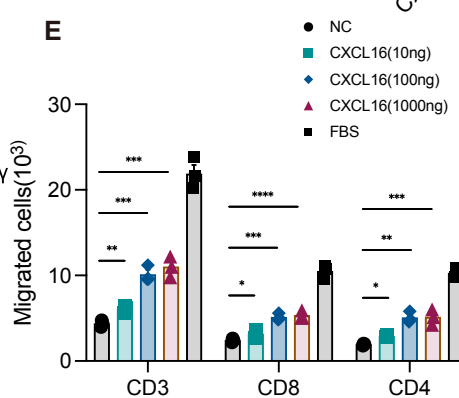

F

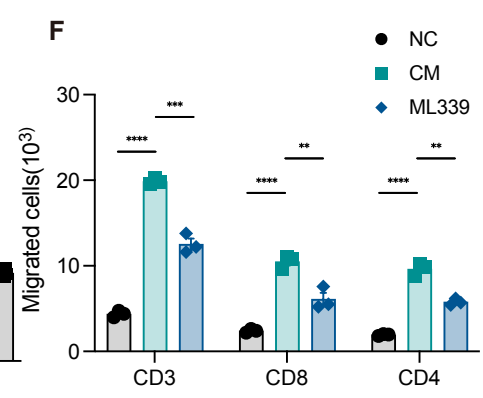

G

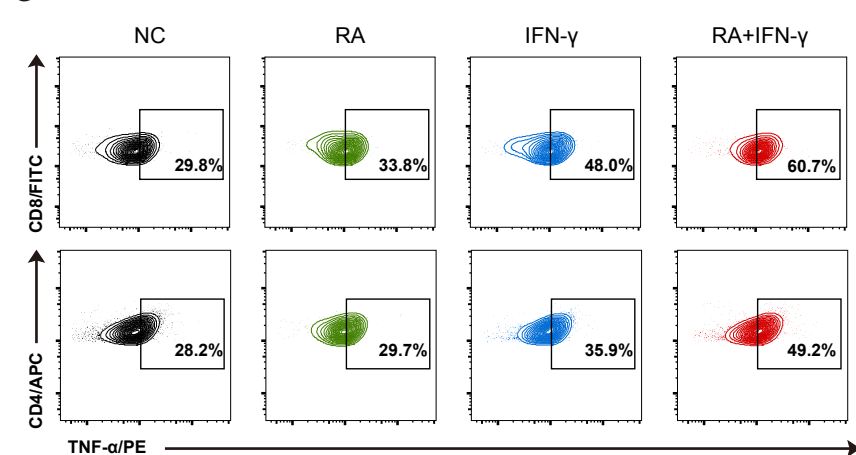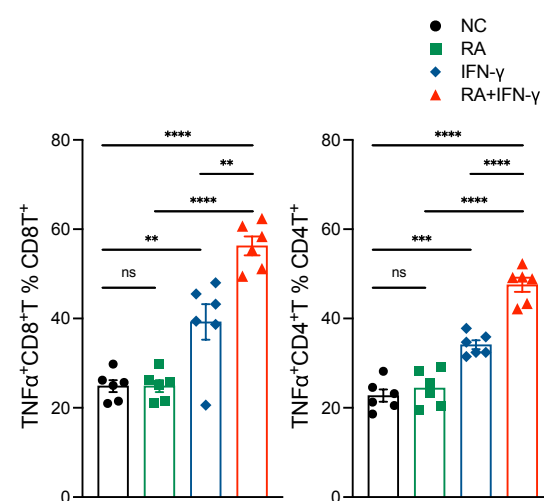

Supplement: Supplementary file 2 — Supporting Information [file ADVS-13-e09340-s001.zip › Mast_Sup9_revised.pdf]

**Supplemental Figure 10**

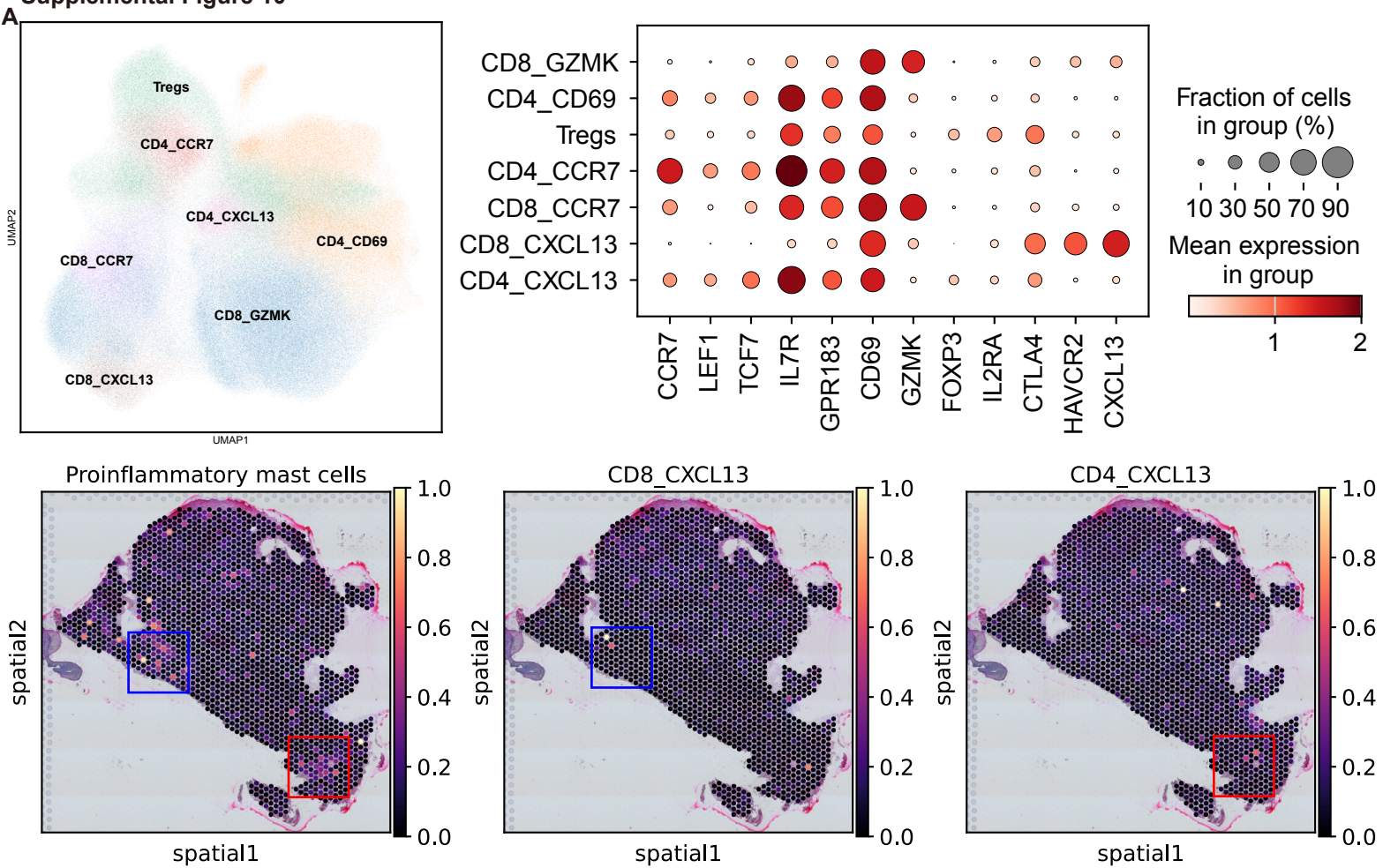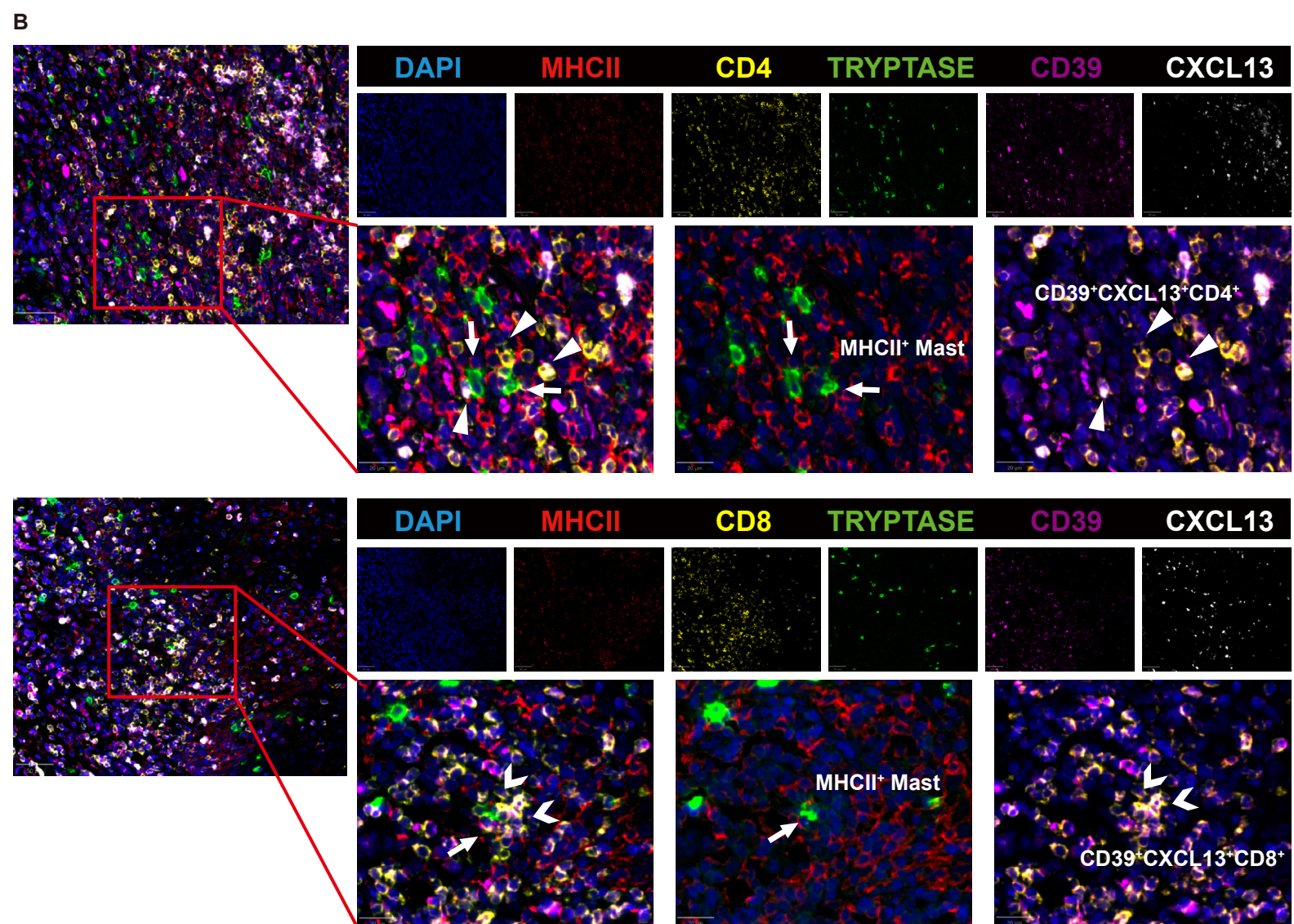

Supplement: Supplementary file 2 — Supporting Information [file ADVS-13-e09340-s001.zip › Mast_Sup10_revised.pdf]

A

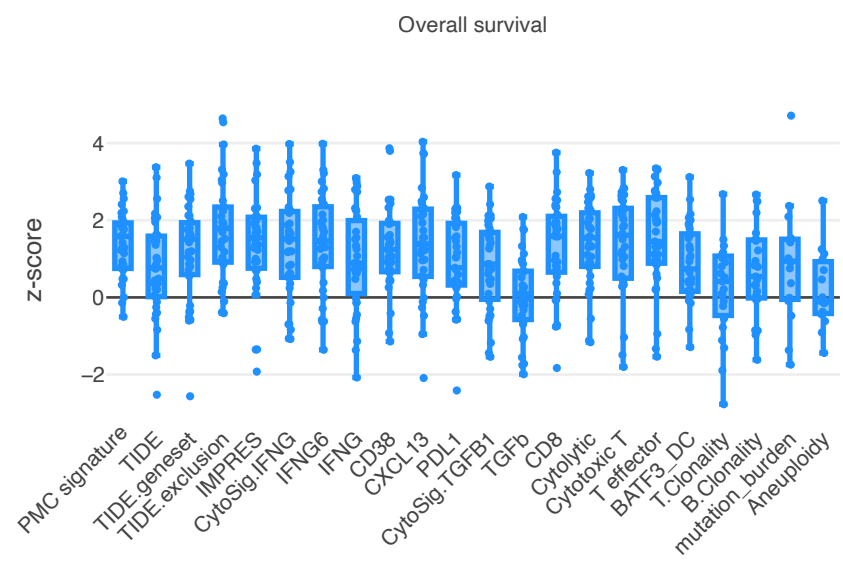

B

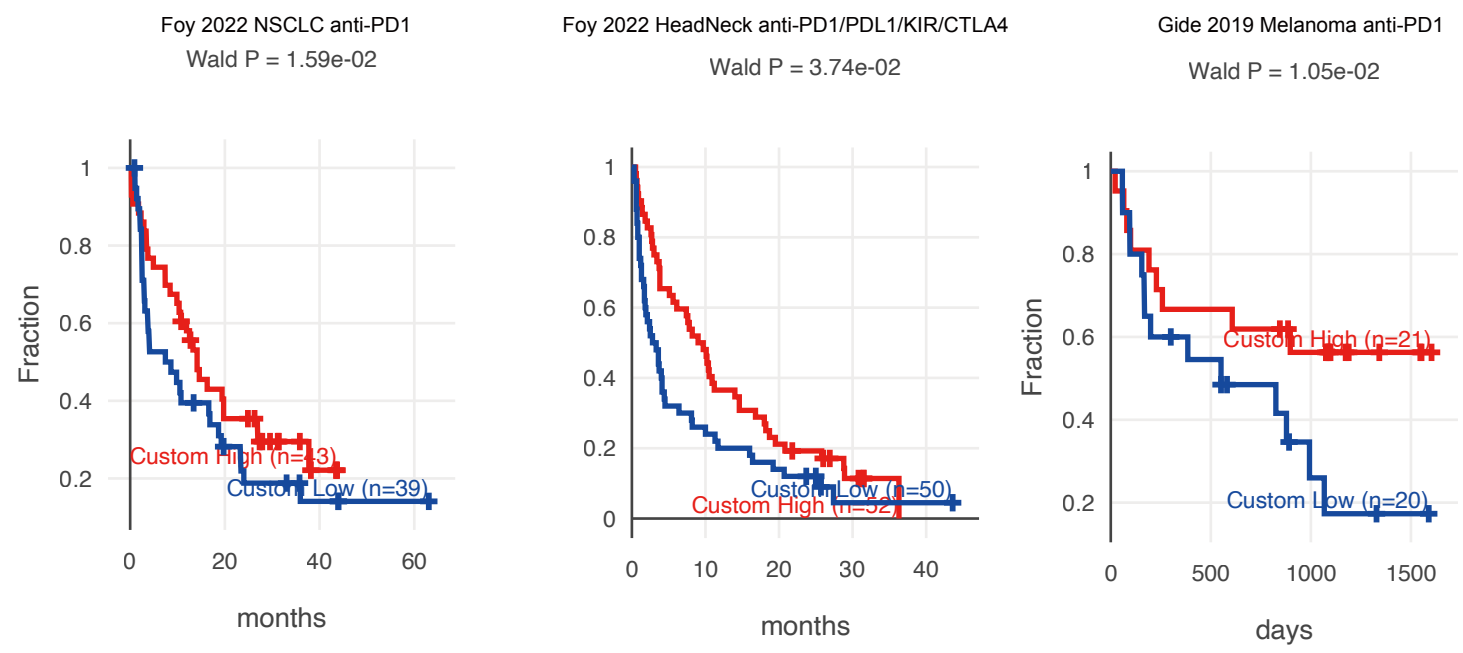

C

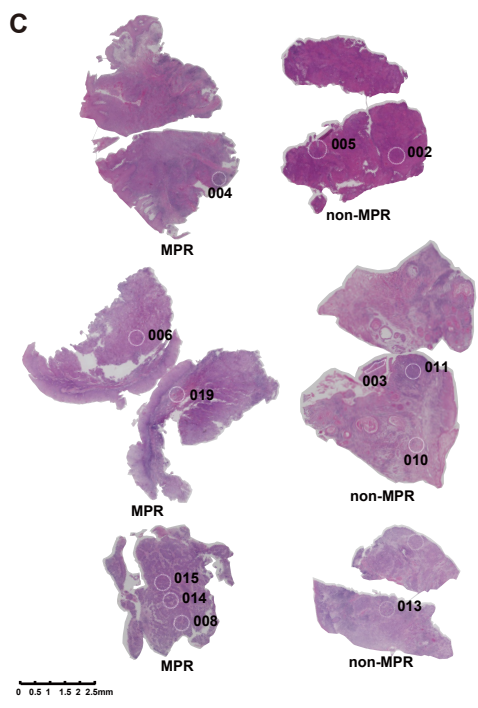

D

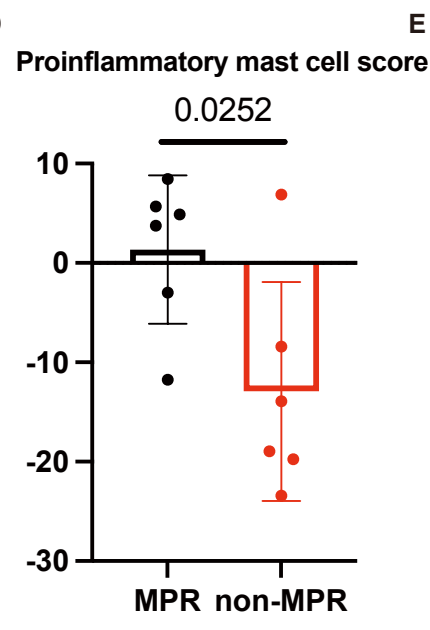

E

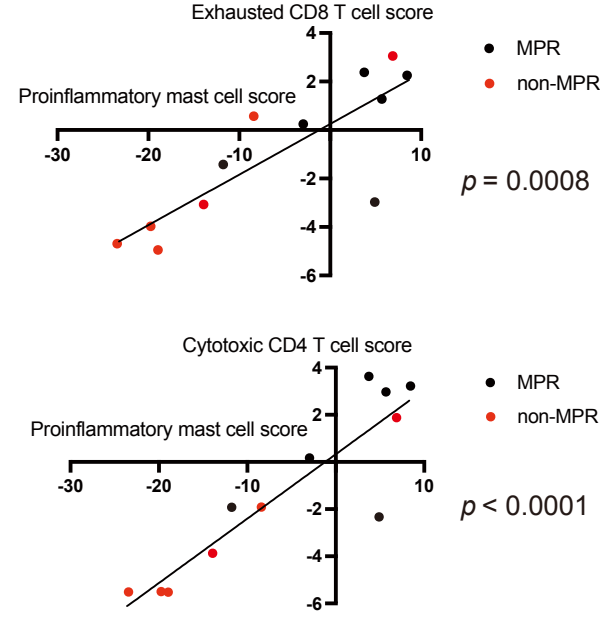

Supplement: Supplementary file 2 — Supporting Information [file ADVS-13-e09340-s001.zip › Mast_Sup11_revised.pdf]

Supplemental Figure 1

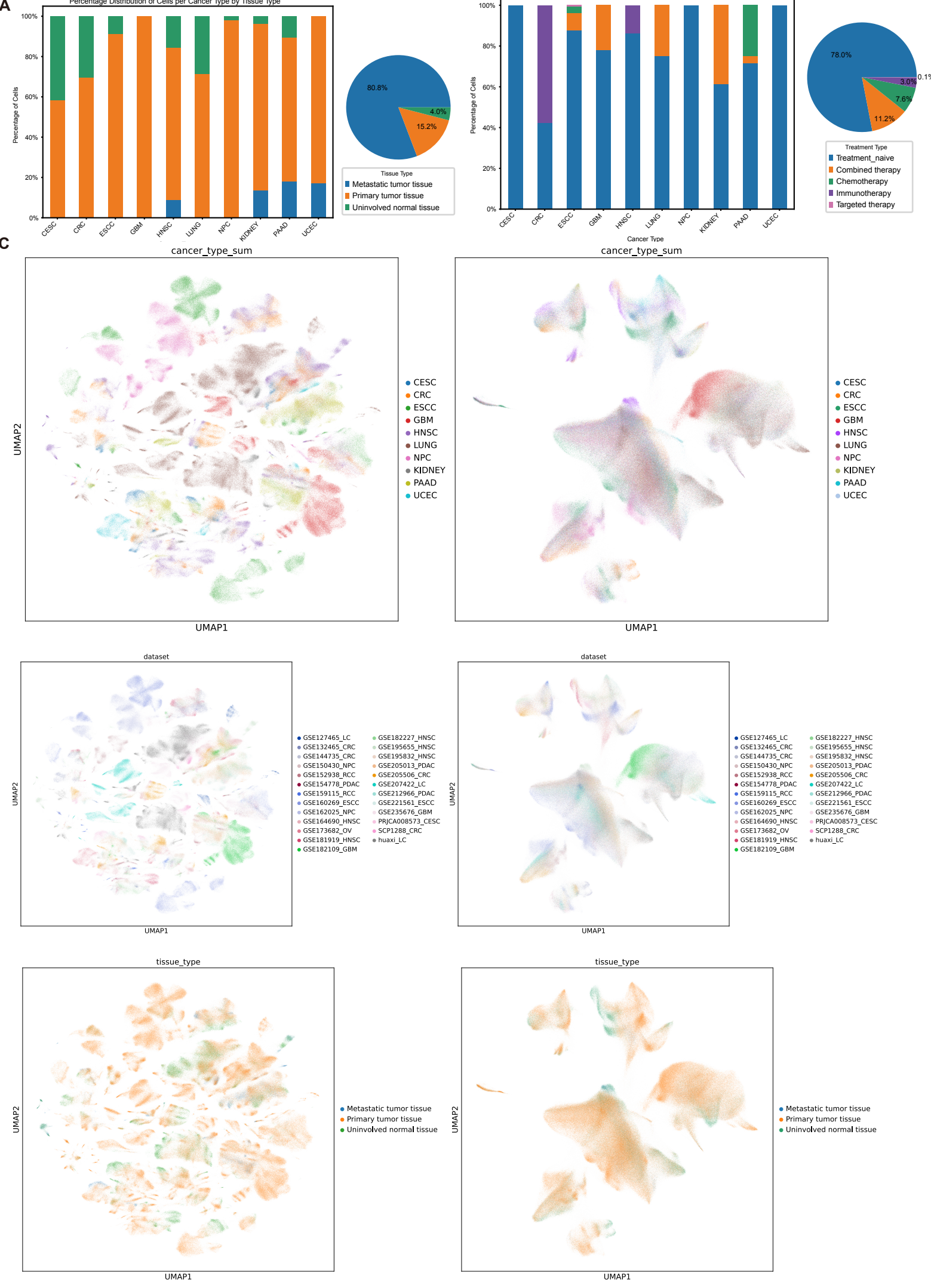

Supplement: Supplementary file 2 — Supporting Information [file ADVS-13-e09340-s001.zip › Mast_Sup1_revised.pdf]

Supplemental Figure 2

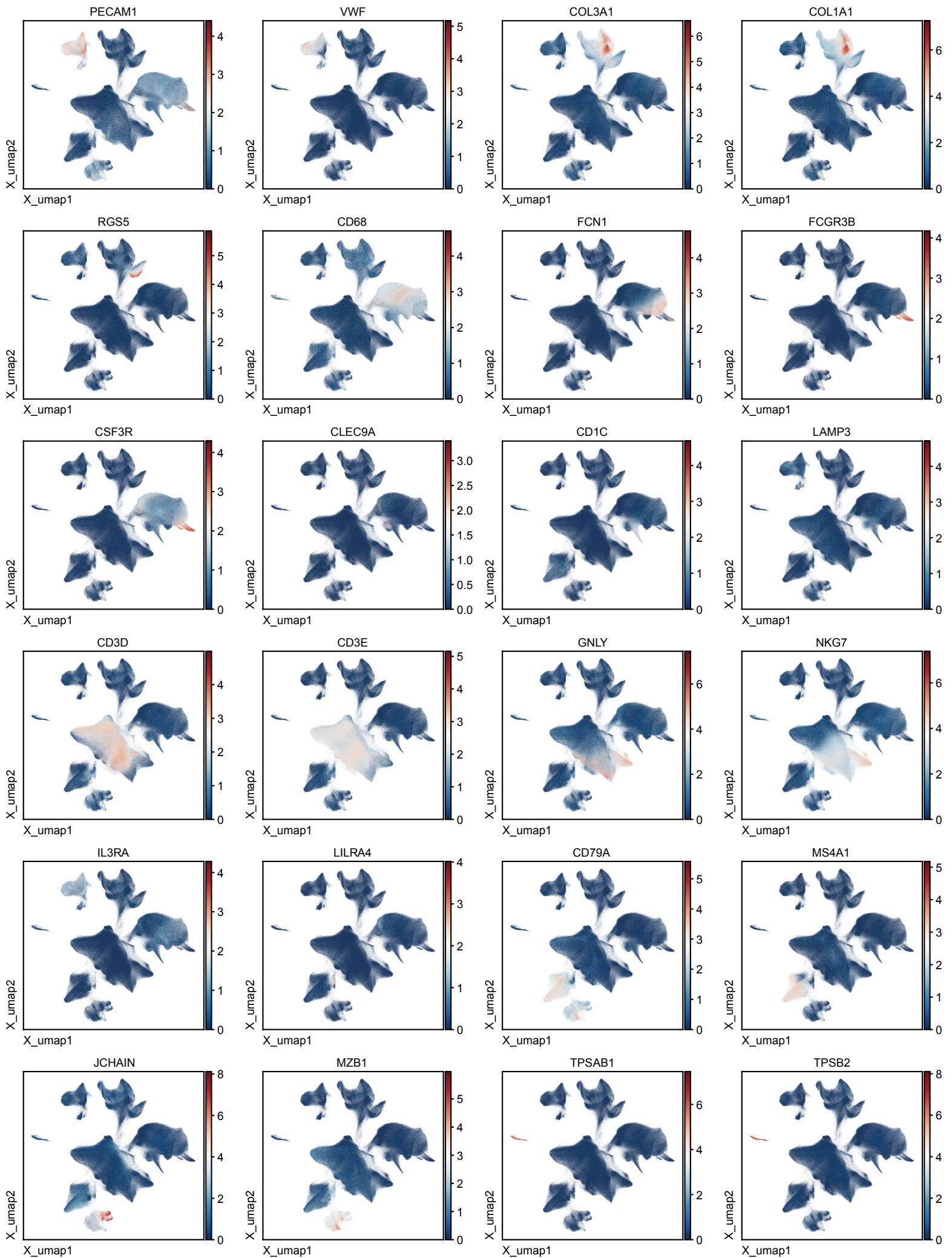

Supplement: Supplementary file 2 — Supporting Information [file ADVS-13-e09340-s001.zip › Mast_Sup2_revised.pdf]

Supplemental Figure 3

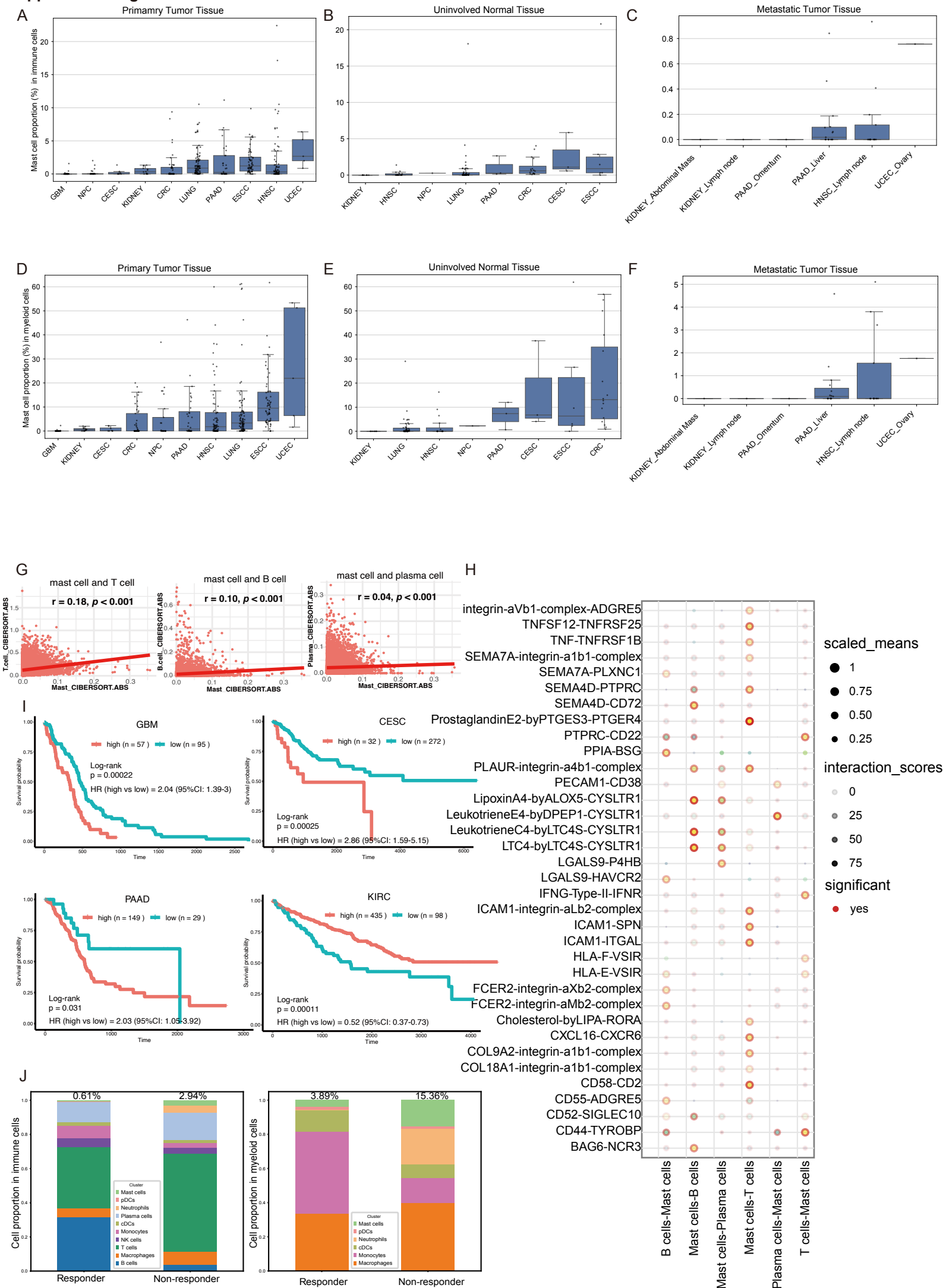

Supplement: Supplementary file 2 — Supporting Information [file ADVS-13-e09340-s001.zip › Mast_Sup3_revised.pdf]

Supplemental Figure 4

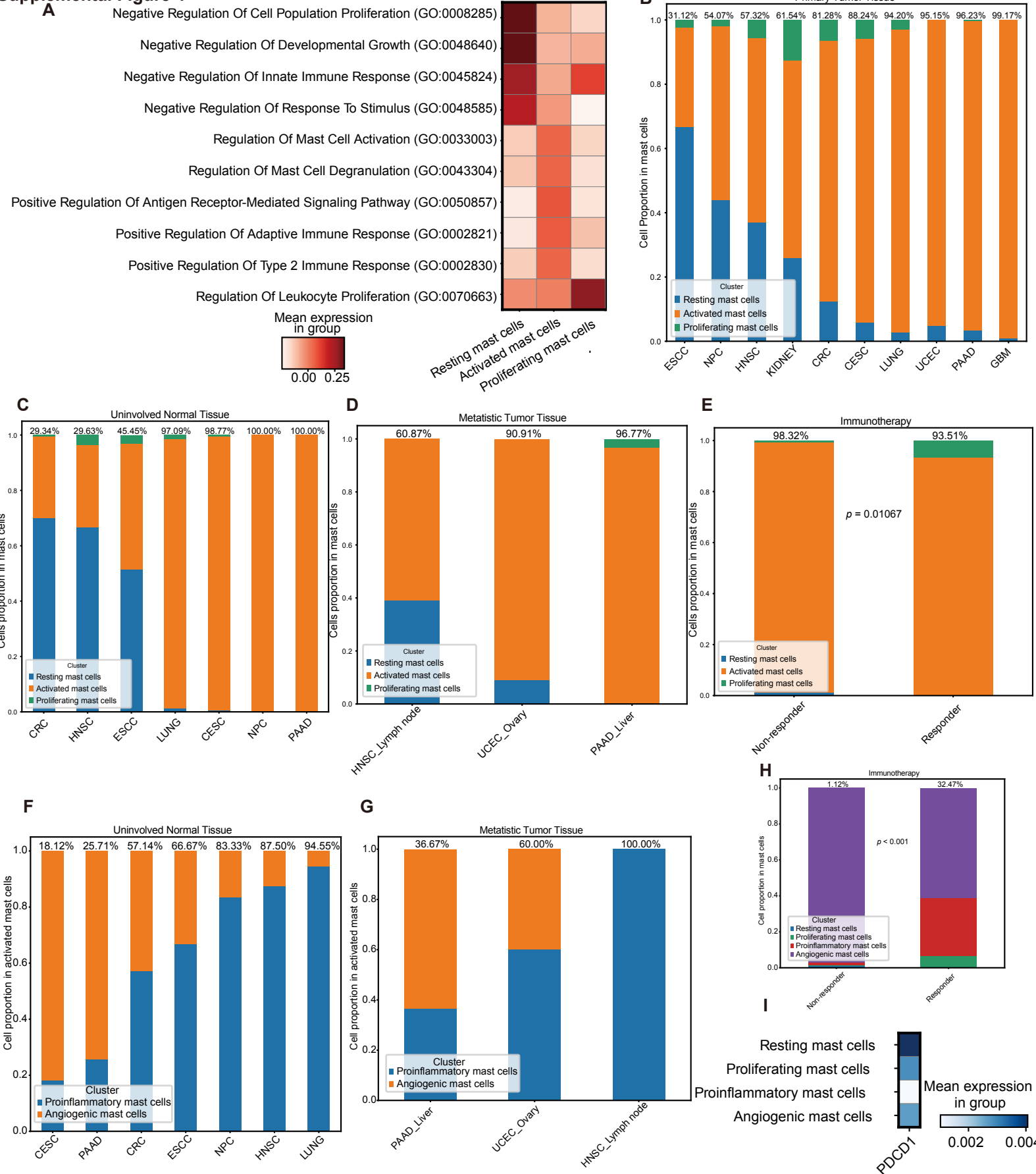

Supplement: Supplementary file 2 — Supporting Information [file ADVS-13-e09340-s001.zip › Mast_Sup4_revised.pdf]
